# Pannexin 3 regulates skin development via Epiprofin

**DOI:** 10.1038/s41598-021-81074-1

**Published:** 2021-01-19

**Authors:** Peipei Zhang, Masaki Ishikawa, Andrew Doyle, Takashi Nakamura, Bing He, Yoshihiko Yamada

**Affiliations:** 1 Molecular Biology Section, National Institute of Dental and Craniofacial Research, National Institutes of Health, Bethesda, MD 20892 USA; 2 Division of Operative Dentistry, Department of Restorative Dentistry, Tohoku University, Graduate School of Dentistry 4-1, Seiryo chou, Aoba-ku, Sendai, Miyagi 980-8575 Japan; 3 Cell Biology Section, National Institute of Dental and Craniofacial Research, National Institutes of Health, Bethesda, MD 20892 USA; 4 Division of Molecular Pharmacology and Cell Biophysics, Department of Oral Biology, Tohoku University Graduate School of Dentistry, Sendai, 980-8575 Japan; 5 Protein Section, Laboratory of Metabolism, Center for Cancer Research, National Cancer Institute, National Institutes of Health, Bethesda, MD 20892 USA

**Keywords:** Cell proliferation, Differentiation, Cell growth, Cell signalling, Cell biology, Developmental biology

## Abstract

Pannexin 3 (Panx3), a member of the gap junction pannexin family is required for the development of hard tissues including bone, cartilage and teeth. However, the role of Panx3 in skin development remains unclear. Here, we demonstrate that Panx3 regulates skin development by modulating the transcription factor, Epiprofin (Epfn). Panx3^−/−^ mice have impaired skin development and delayed hair follicle regeneration. Loss of Panx3 in knockout mice and suppression by shRNA both elicited a reduction of Epfn expression in the epidermis. In cell culture, Panx3 overexpression promoted HaCaT cell differentiation, cell cycle exit and enhanced Epfn expression. Epfn^−/−^ mice and inhibition of Epfn by siRNA showed no obvious differences of Panx3 expression. Furthermore, Panx3 promotes Akt/NFAT signaling pathway in keratinocyte differentiation by both Panx3 ATP releasing channel and ER Ca^2+^ channel functions. Our results reveal that Panx3 has a key role factor for the skin development by regulating Epfn.

## Introduction

The skin is the largest organ of the human body, acting as the first line of defense from external factors with protective, immunologic, metabolism, thermoregulatory, and sensory functions^1^. Skin integrity and its normal function depend on the ability of keratinocytes to form the permeability barrier of the stratum corneum^2^. The epidermis contains five distinct layers: 1) basal, 2) spinosum, 3) granulosum, 4) lucidum and 5) corneum layers. Layers one through four are formed through the sequential differentiation of keratinocytes. The maturation process of keratinocytes has an integral relationship with a gradual amplification in calcium from basal layer of stem cells and proliferation progenitor cells to the outermost cornified layer containing terminally differentiated keratinocytes^3^. Calcium plays a well-known critical role in barrier function repair and skin homeostasis by regulating keratinocytes proliferation and differentiation^4^.

Maintenance of homeostasis in the skin requires direct intracellular and cell-extracellular communication^5^. Gap junction proteins play an important role in cell communications through their function as channels, allowing the exchange of various molecules, such as ions, metabolites, second messengers, and morphogens between adjoining cells^6,7^. There are two main families of gap junction proteins in vertebrates, connexins (Cxs) and pannexins (Panxs)^8^ with the latter being comprised of three family members, Panx1, 2 and 3^9^. Panx1 is ubiquitously expressed in most tissues, while Panx2 appears to be more restricted to the central nervous system^10^. Panx3 is highly expressed in osteoblasts, synovial fibroblasts, bones, cartilage, teeth and skin^9,11,12^ and is found to cell differentiation and cell cycle exit of preosteoblast and chondrocyte^13–15^. Loss of Panx3 in mice lead to severe skeletal abnormalities^16,17^. Panx3 acts as a channel having different functions depending on its localization in the cell. At the plasma membrane, Panx3 releases intracellular ATP into the extracellular space, to activate PI3K/Akt pathway. This phosphorylation of Akt initiates endoplasmic reticulum (ER)-associated Panx3 to increase intracellular Ca^2+^ levels, which upregulates the calmodulin/calcineurin/NFAT signaling pathway to promote differentiation^11,14,18^. Panx3 deficiency delays wound healing in the skin by reducing epithelial–mesenchymal transition, keratinocytes migration, and collagen remodeling^19^. However, the functions of Panx3 in skin tissue development remain poorly understood.

Epiprofin (Epfn), also known as Sp6, is a zinc finger transcription factor that functions to promote both the proliferation and differentiation of keratinocytes^20^. Epfn^−/−^ mice display defects in tooth morphology, supernumerary tooth formation, digit fusion in the limbs, skin abnormalities and hairlessness^20–24^. In cell culture, low Epfn expression promotes proliferation of keratinocyte progenitors, whereas high Epfn expression promotes cell cycle exit and induces differentiation through the activation of Notch1^20^.

In this study, we show that Panx3 mediates keratinocyte differentiation by regulating Epfn. We find a thin epidermis in Panx3^−/−^ mice and reduced Epfn expression in skin. In HaCaT cells, Panx3 overexpression increases Epfn expression and keratinocyte differentiation, while shRNA ablation of Panx3 decreases the presence of Epfn. In contrast, Epfn deletion does not interfere with Panx3 expression in skin and keratinocyte differentiation, suggesting that Panx3 is an upstream regulatory molecule of Epfn during skin development. Further, Panx3 promotes keratinocyte differentiation via Epfn signaling through the activation of Akt/NFAT signaling by its ATP releasing channel and ER Ca^2+^ channel functions.

## Results

### Panx3^−/−^ mice show a thin epidermis and delayed hair follicle regeneration

**Figure 1:**
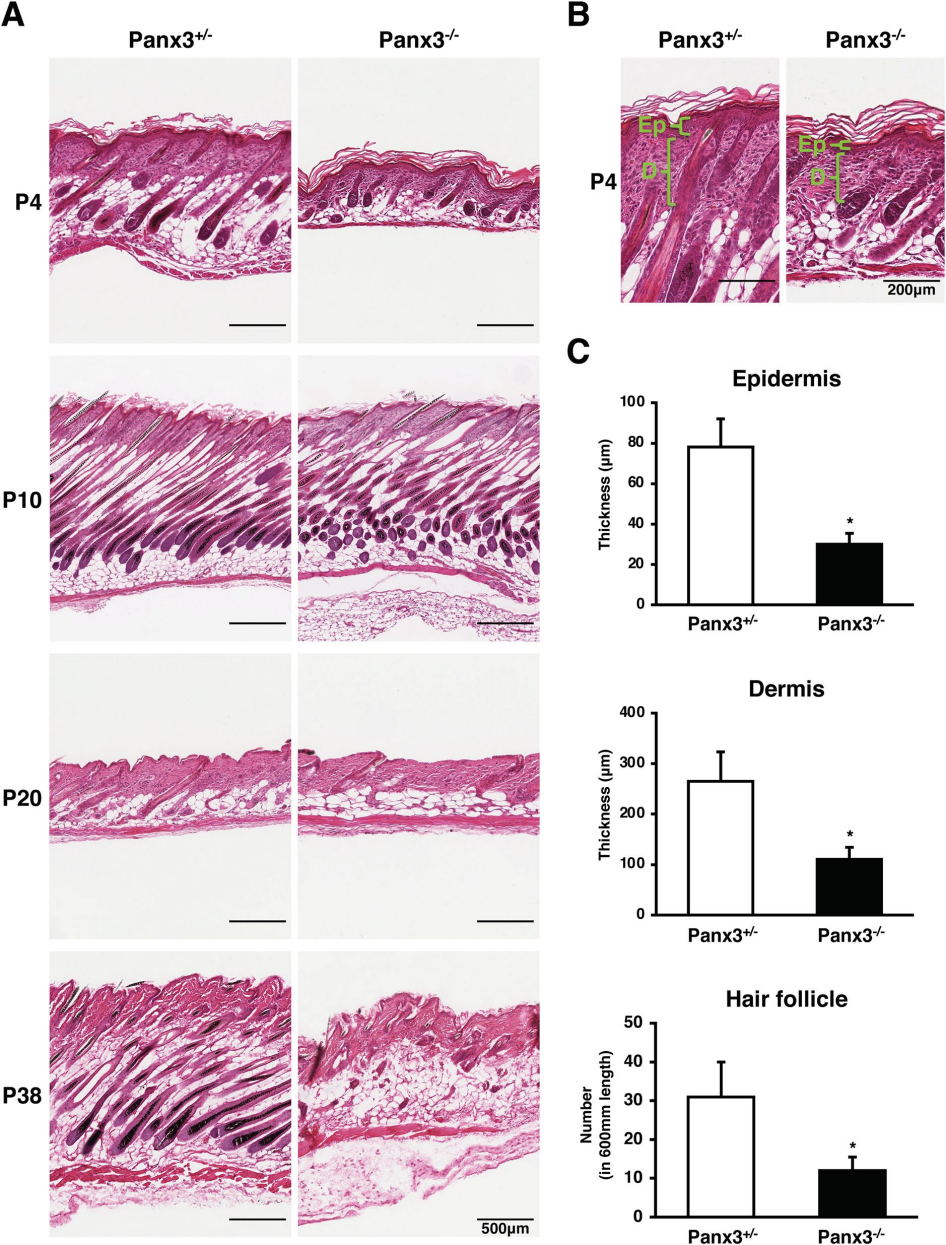
Panx3 regulates skin structure and hair follicle cycle. (**A**) Representative histology images (H&E staining) of Panx3^+/−^ and Panx3^−/−^ mice skin on different postnatal days. (**B**) Higher magnification H&E stained images of Panx3^+/−^ and Panx3^−/−^ mice skin on P4. Ep: epidermis; D: dermis. (**C**) Measurements of epidermis and dermis thickness and hair follicle numbers in Panx3^+/−^ and Panx3^−/−^ mice on P4. *p < 0.01. Error bars represent the mean ± SD; N = 3.

To elucidate the role of Panx3 in the skin, we first compared histological cross-sections of the skin in Panx3^+/−^ and Panx3^−/−^ mice at multiple post-natal ages (Fig. 1A; Supplementary Fig. 1). The skin phenotype between Panx3^+/+^ and Panx3^+/−^ mice were comparable and was used as a control (Supplementary Fig. 2). On postnatal day four (P4), Panx3^−/−^ mice showed less pigmentation (Supplementary Fig. 1), a thinner epidermis and dermis than Panx3^+/−^ mice (Fig. 1B). The number of hair follicles in Panx3^−/−^ skin was lower than that of Panx3^+/−^ skin (Fig. 1A,C). Further examination revealed Panx3^−/−^ mice showed no obvious difference in the first hair cycle and no premature hair follicle anagen cessation, hair loss, or hair thinning commonly associated with severe malnutrition (Fig. 1A, P4 and P10). However, in the second hair cycle from five to eight weeks old, Panx3^−/−^ mice demonstrated a delayed hair follicle cycle with a prolonged time in the telogen (quiescent) phase (Fig. 1A, P20 and P38; Supplementary Fig. 1).

### Panx3^−/−^ mice show the inhibition of keratinocyte differentiation

**Figure 2:**
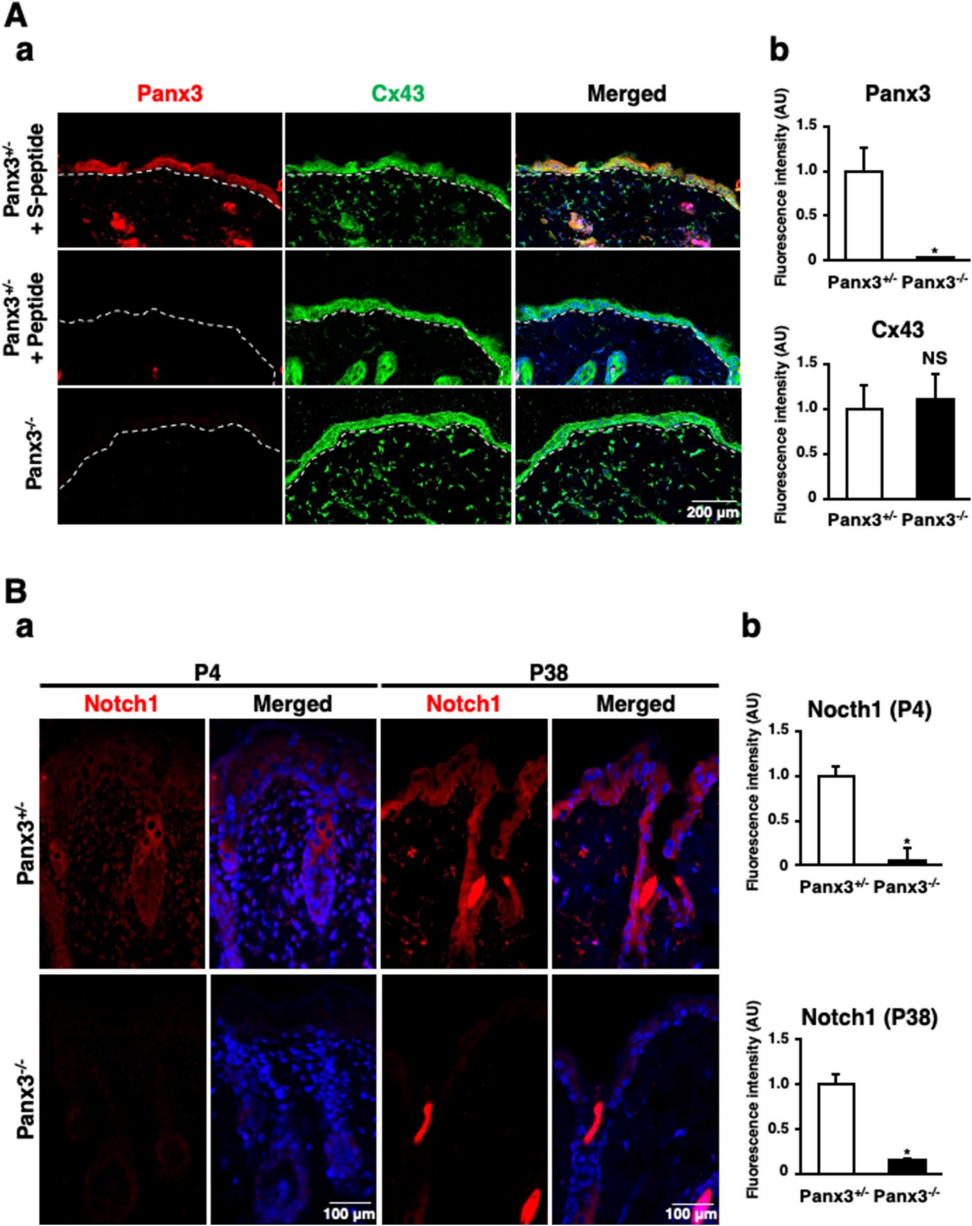
Panx3 deficiency inhibits keratinocyte differentiation. (**A**) Immunostaining of skin of 6 weeks old Panx3^+/−^ (top and middle) and Panx3^−/−^ (bottom) mice for to Panx3 (red) and Cx43 (green) in the presence (top and middle) or absence (bottom) of Panx3 cognate peptide (middle) or control peptide (S-peptide) (top) (**a**). Dotted lines indicate borders between epidermis and dermis. (**B**) Immunostaining of skin of Panx3^+/−^ and Panx3^−/−^ mice on P4 and P38 with antibody to Notch1(red). The nuclei were counterstained with DAPI (blue in merged) (**a**). Relative fluorescence intensity comparison of images were analyzed (M&M) (**A**,**b** and **B**,**b**). *p < 0.01. Error bars represent the mean ± SD; N = 3.

We next investigated the localization of Panx3 within the epidermis*.* In Panx3^+/−^ mice, Panx3 was expressed throughout the epidermis from the basal layer to the granular layer, as well as the corneal layer in the skin at adult stage (Fig. 2A, a, left panel in upper lane). Panx3 immunostaining was effectively eliminated when the anti-Panx3 antibody was mixed with its cognate peptide (Fig. 2A, a, left panel in middle lane). Cx43, which is ubiquitously expressed in skin^25^, was not abolished in Panx3 ^−/−^ mice (Fig. 2A, a and b, middle panel in bottom lane).

Since Panx3^−/−^ mice showed an inability to regulate keratinocyte differentiation, we next observed the expression of keratinocyte differentiation marker, Notch1, at P4 and P38. Unlike Panx3^+/−^ mice, we found no evidence of Notch1 expression in the epidermis layer at either P4 and P38 of Panx3^−/−^ mice, (Fig. 2B, a and b). These results suggest that Panx3 regulates keratinocyte differentiation.

### Panx3 promotes HaCaT cell differentiation and inhibits cell proliferation

**Figure 3:**
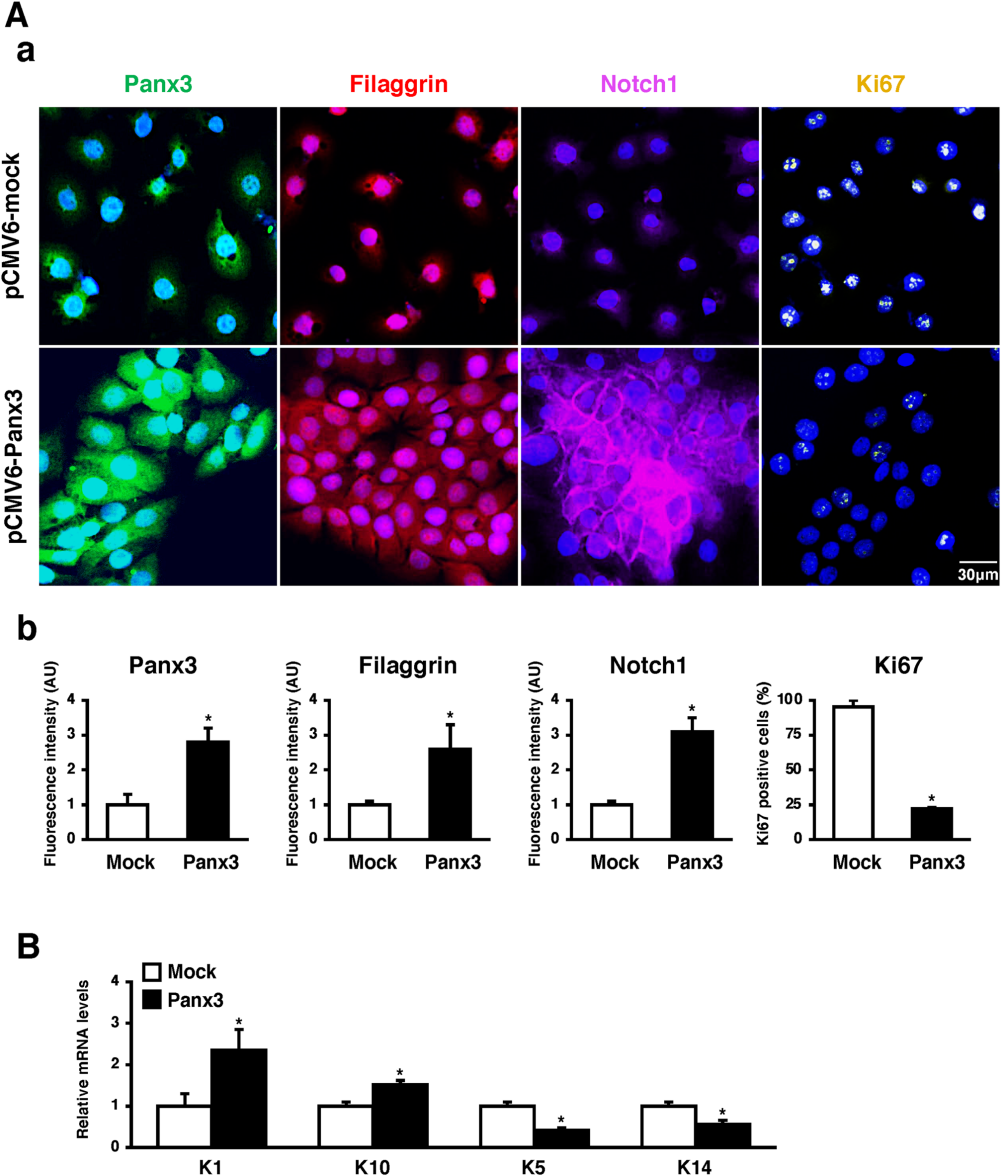
Panx3 regulates HaCaT cell differentiation. (**A**) Immunostaining of Panx3 (green), Filaggrin (red), and Notch1 (purple), Ki67 (yellow) in pCMV6-mock or pCMV6-Panx3 transfected HaCaT cells cultured in normal condition media (**a**). Relative fluorescence intensity comparison of images and % of Ki67 positive cells were analyzed (**b**). *p < 0.01. Error bars represent the mean ± SD; N = 3. The nuclei were counterstained with DAPI (blue in merged). (**B**) qPCR for K1, K10, K5, and K14 in pCMV6-mock or pCMV6-Panx3 transfected HaCaT cells. Results represent the mean ± SD; N = 3. *p < 0.01.

**Figure 4:**
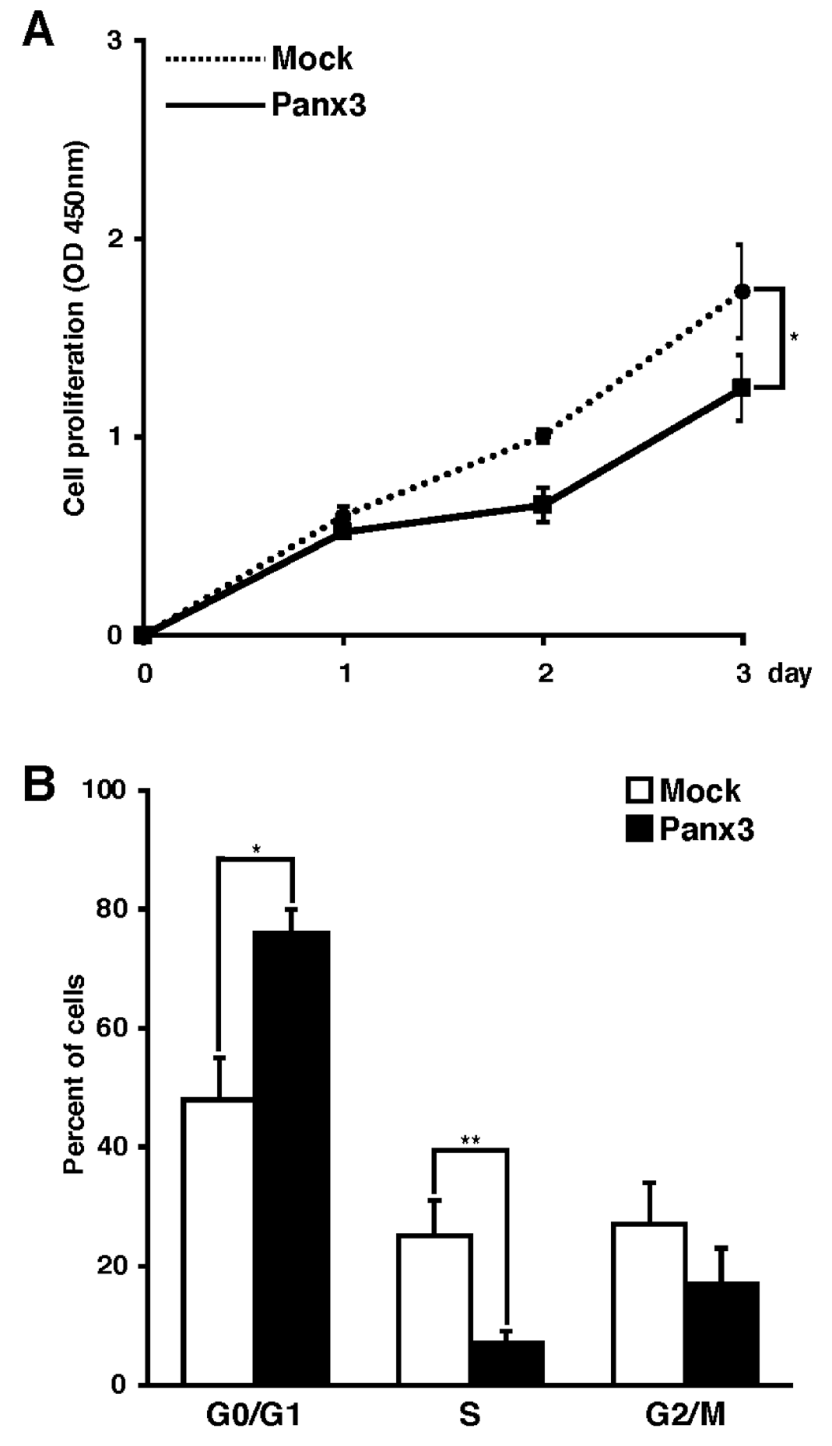
Panx3 promotes cell cycle exit in HaCaT cell proliferation. (**A**) pCMV6-mock or pCMV6-Panx3 were transiently transfected HaCaT cells were cultured in normal condition media for the indicated days. *p < 0.01. Error bars represent the mean ± SD; N = 3. (**B**) FACS analysis of cell cycle on pCMV6-mock or pCMV6-Panx3 transiently transfected HaCaT cells cultured in normal condition media for 3 days. The cells were stained with propidium iodide, and cell cycle stages were measured by FACS analysis. The panels represent distribution of cells (%) in the G0/G1, S, and G2/M phases. **p < 0.05, *p < 0.01. Error bars represent the mean ± SD; N = 3.

We next analyzed Panx3 function in keratinocyte differentiation using the human immortalized keratinocyte cell line, HaCaT cells. HaCaT cells normally are maintained in calcium-free keratinocyte media (KM) and can be induced to differentiate through the addition of high Ca^2+^ concentration in the media (KM + high Ca^2+^). We also used an overexpression model (pCMV6-Panx3) and shPanx3 to further establish the influence of Panx3 on keratinocyte differentiation (Supplementary Fig. 3). Panx3 overexpression increased the expression of the terminal keratinocyte differentiation marker Filaggrin and Notch1, while showing decreased Ki67, a marker for cell proliferation (Fig. 3A, a and b). Moreover, quantitative RT-PCR (qPCR) analysis showed the differentiation markers, *K1*, and *K10* were increased in Panx3 overexpressed cells, while immature markers, *K5*, and *K14* were decreased (Fig. 3B). In addition, Panx3 overexpression inhibited HaCaT cells proliferation (Fig. 4A), while promoting cell cycle arrest at G0/G1 phase (Fig. 4B; Supplementary Fig. 4). Together, these results indicate that Panx3 promotes keratinocyte differentiation and inhibits cell proliferation.

### Panx3 regulates Epfn in keratinocyte differentiation

**Figure 5:**
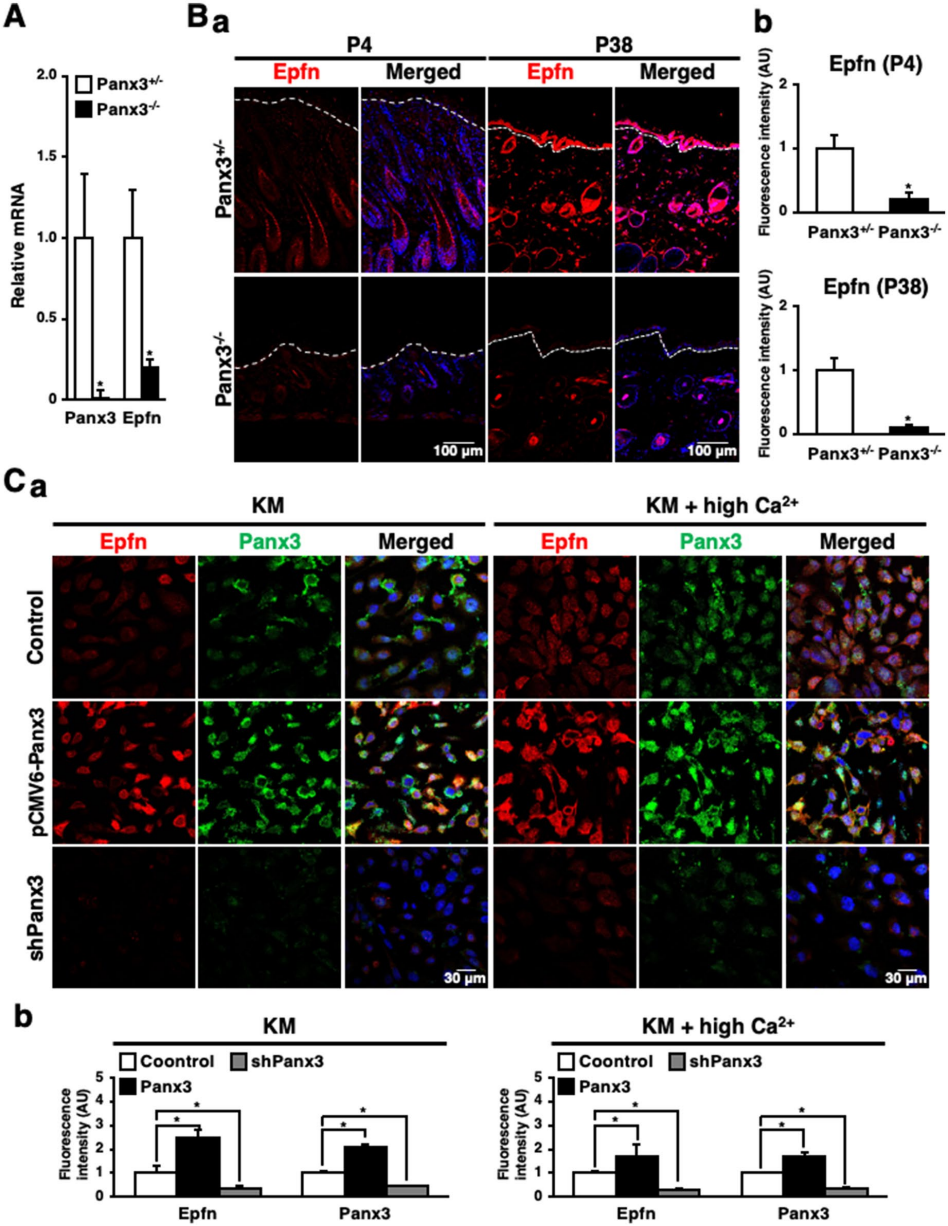
Panx3 regulates Epfn expression during keratinocyte differentiation. (**A**) qPCR for Panx3 and Epfn using total RNA prepared from skin of 6 weeks old Panx3^+/−^ and Panx3^−/−^ mice. Results represent the mean ± SD; N = 3. *p < 0.01. (**B**) Immunostaining of Epfn (red) in Panx3^+/−^ and Panx3^−/−^ mice skin of P4 and P38. The nuclei were counterstained with DAPI (blue in merged) (**a**). Relative fluorescence intensity comparison of images were analyzed (**b**). *p < 0.01. Error bars represent the mean ± SD; N = 3. (**C**) Immunostaining for Panx3 (green) and Epfn (red) in pCMV6-Panx3 or shPanx3 transiently transfected HaCaT cells cultured in normal medium (KM) and differentiation medium containing 1.4 mM Ca^2+^ (KM + high Ca^2+^). The nuclei were counterstained with DAPI (blue in merged) (**a**). Relative fluorescence intensity comparison of images were analyzed (**b**). *p < 0.01. Error bars represent the mean ± SD; N = 3.

Keratinocyte development is notably modulated by a zinc finger transcription factor, Epiprofin (Epfn)^20^. Low Epfn expression promotes HaCaT cell proliferation. High Epfn expression shifts the cell fate from proliferation to differentiation by activating the Notch signaling pathway^20^. Thus, we hypothesed that Epfn could be involved in Panx3 mediated keratinocyte differentiation. We first compared Epfn expression in skin between Panx3^+/−^ and Panx3^−/−^ mice by qPCR. Epfn expression was significantly decreased in Panx3^−/−^ mice (Fig. 5A). We next observed Epfn expression and localization in skin of both Panx3^+/−^ and Panx3^−/−^ mice by immunohistochemistry. Panx3^+/−^ mice revealed that Epfn was expressed in hair follicles including inner- and outer- root sheath cells at P4 as well as in both the epidermis and within hair follicle keratinocytes at P38. In contrast, Epfn was significantly reduced in Panx3^−/−^ mice at both ages (Fig. 5B, a and b). In addtion, our HaCaT model demonstrated a similar high expression of Epfn in Panx3 overexpressing cells, while Epfn was reduced in shPanx3 cells (Fig. 5C, a and b). During differentiation condition (KM + high Ca^2+^), both Panx3 and Epfn demonstrated a similar expression pattern in control HaCaT cells compared with those in normal media (KM) (Fig. 5C, a and b). In KM + high Ca^2+^ condition, proliferation marker, K14 was reduced, while differentiation markers, Filaggrin, K10 and Panx3 were increased (Supplementary Fig. 5A). Further, shPanx3 reduced Panx3 and differentiation marker, Notch1 expression in KM + Ca^2+^ condition (Supplementary Fig. 5B). These results indicate that Panx3 regulates Epfn expression during keratinocyte differentiation.

### Epfn deficiency doesn’t interfere with Panx3 expression

**Figure 6:**
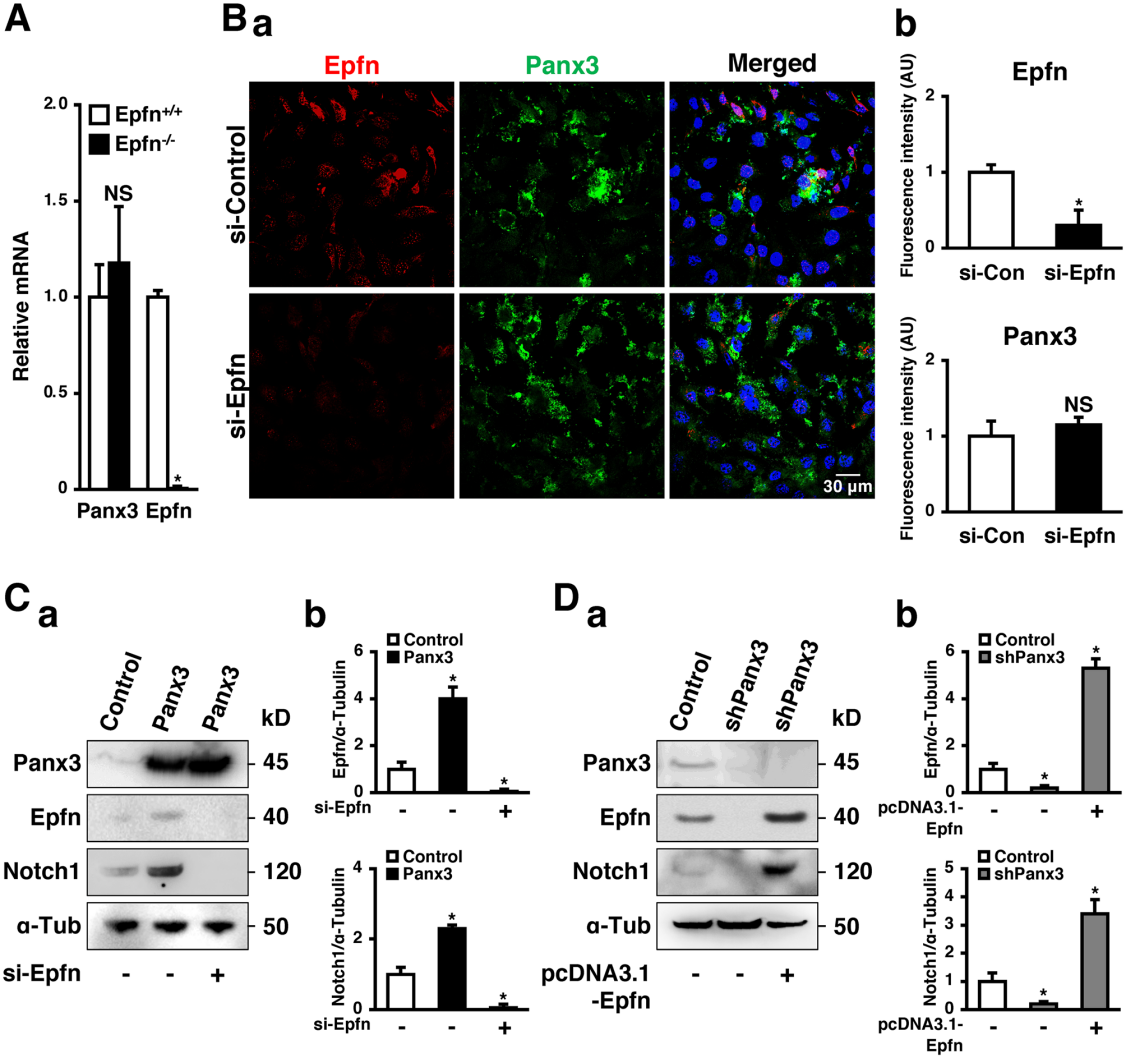
Epfn deficiency does not interfere with Panx3 expression. (**A**) qPCR for Panx3 and Epfn using total RNA prepared from skin of 6 weeks old Epfn^+/+^ and Epfn^−/−^ mice. Results represent the mean ± SD; N = 3. *p < 0.01. NS, not significant. (**B**) Immunostaining of Panx3 (green) and Epfn (red) in si-Control or si-Epfn transfected HaCaT cells cultured in normal medium. The nuclei were counterstained with DAPI (blue in merged) (**a**). Relative fluorescence intensity comparison of images were analyzed (**b**). (**C**) Western blots of Epfn and Notch1 in Panx3 overexpressed HaCaT cells transfected with or without si-Epfn (**a**). Quantification of the ratios of Epfn/α-Tubulin (upper panel) and Notch1/α-Tubulin (lower panel). (**D**) Western blots of Epfn and Notch1 in shPanx3 transfected HaCaT cells with or without pcDNA3.1-Epfn (**a**). Quantification of the ratios of Epfn/α-Tubulin (upper panel) and Notch1/α-Tubulin (lower panel). *p < 0.01. NS, not significant.

We further asked whether Epfn modulates Panx3 expression in keratinocyte differentiation. Q-PCR with skin of Epfn^+/+^ or Epfn^−/−^ mice showed that Panx3 expression was comparable (Fig. 6A). Moreover, Panx3 expression was comparable in HaCaT cell with si-Control or si-Epfn by immunostaining (Fig. 6B, a and b). These results suggest that Epfn is not required for Panx3 expression and is likely downstream of its cellular function. For further confirmation that Epfn is a downstream molecule of Panx3 during keratinocyte differentiation, we analyzed Notch1, a downstream target of both Epfn^20^ and Panx3 (Figs. 2B and 3A) by si-Epfn and Epfn overexpression (pcDNA3.1-Epfn)^20,23^ into Panx3 overexpressed and shPanx3 transfected HaCaT cells, respectively (Fig. 6C,D). si-Epfn reduced Notch1 expression in Panx3 overexpressed cells, which normally upregulated Notch1 expression (Fig. 6C, a and b), while Epfn overexpression increased Notch 1 in shPanx3 transfected cells, which downregulated Notch1 (Fig. 6D, a and b). Thoes results indicate that Epfn is downstream of Panx3 during keratinocyte differentiation.

### Panx3 regulates HaCaT differentiation via Akt/NFAT signaling

**Figure 7:**
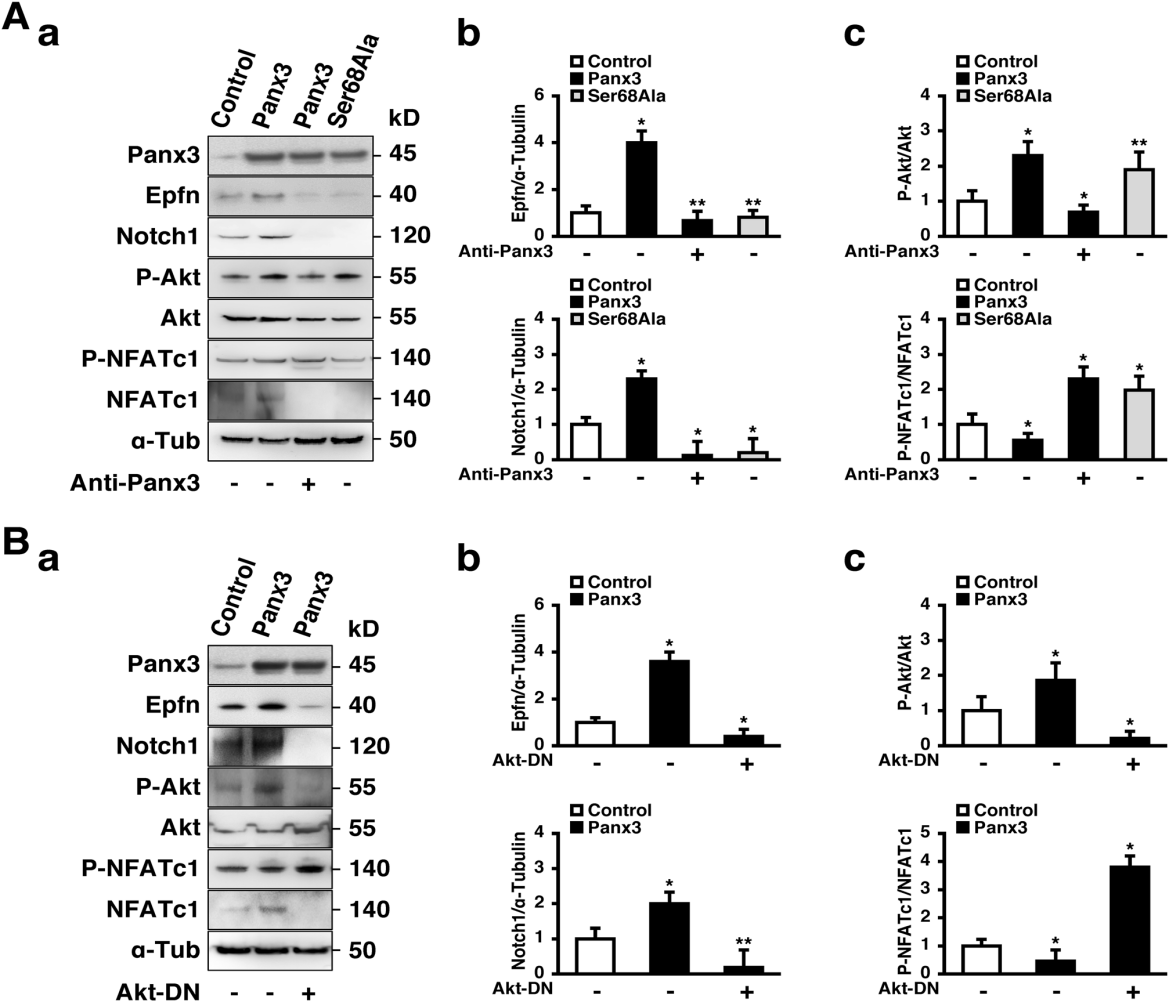
Panx3 ER Ca^2+^ channel regulates Epfn via Akt/NFAT signaling pathway. HaCaT cells were cultured for 3 days in normal media. Western blotting was performed with antibodies to Panx3, Epfn, Notch1, phospho-Akt (P-Akt), Akt, phospho-NFATc1 (P-NFATc1), NFATc1, and α-Tubulin. The cropped blots were used in the figures. Full blot is shown in the Supplementary Information (Full Original Blots-I–IV). (**B**) Quantification of the ratios of Epfn/α-Tubulin (upper panel of **A**,**b** and **B**,**b**) and Notch1/α-Tubulin (lower panel of **A**,**b** and **B**,**b**), P-Akt/Akt (upper panel of **A**,**c** and **B**,**c**) and P-NFATc1/NFATc1 (lower panel of **A**,**c** and b,**c**). **p < 0.05, *p < 0.01. Error bars represent the mean ± SD; N = 3.

We next asked which signaling pathways are involved in Panx3-mediated keratinocyte differentiation through Epfn. Previously, we reported that Panx3 functions as ATP releasing channel and ER Ca^2+^ channel in HaCaT cell^19^. Thus, we hypothesized that Panx3 mediated ATP and Ca^2+^ signaling could be involved. To answer this question, we analyzed the activation of Akt and NFAT in HaCaT cells by Western blotting (Fig. 7). Panx3 overexpression promoted phosphorylation of Akt and the level of dephosphorylated NFATc1 (active form) (Fig. 7A,B). We also found Epfn and Notch1 were also increased by Panx3 overexpression. To analyze how Panx3 channel functions are involved, we first applied Panx3 antibody to block Panx3 ATP at the cell membrane^11,14^. Application of Panx3 antibody inhibited both Akt and NFATc1 activation in Panx3 overexpressed cells and therefore decreased Epfn and Notch1 expression. To test the 2nd function of Panx3 as an ER Ca^2+^ channel mediated by ATP/PI3K/Akt signals^14,19^, a mutation was introduced to Panx3 at serine 68 (Ser68Ala) . to impair Panx3 ER Ca^2+^ release^18^. Ser68Ala inhibited the activation of NFATc1 and both Epfn and Notch1 expression (Fig. 7A, a, b and c). These results suggest that Panx3 as an ER Ca^2+^ channel strongly regulates Epfn expression through NFATc1/Ca^2+^ signaling for keratinocyte differentiation. Further, since Panx3 ER Ca^2+^ channel is activated by Akt signaling^14,18,19^, we investigated Epfn expression and NFATc1 activation with Akt dominant negative vector (Akt-DN) to confirm whether Panx3 ER Ca^2+^ channel function is critical for Epfn expression. Akt-DN inhibited NFATc1 activation and decreased Epfn and Notch1 expression (Fig. 7B, a, b and c). These results indicate that Panx3 regulates Epfn expression through Panx3 ER Ca^2+^ channel function during keratinocyte differentiation.

## Discussion

**Figure 8:**
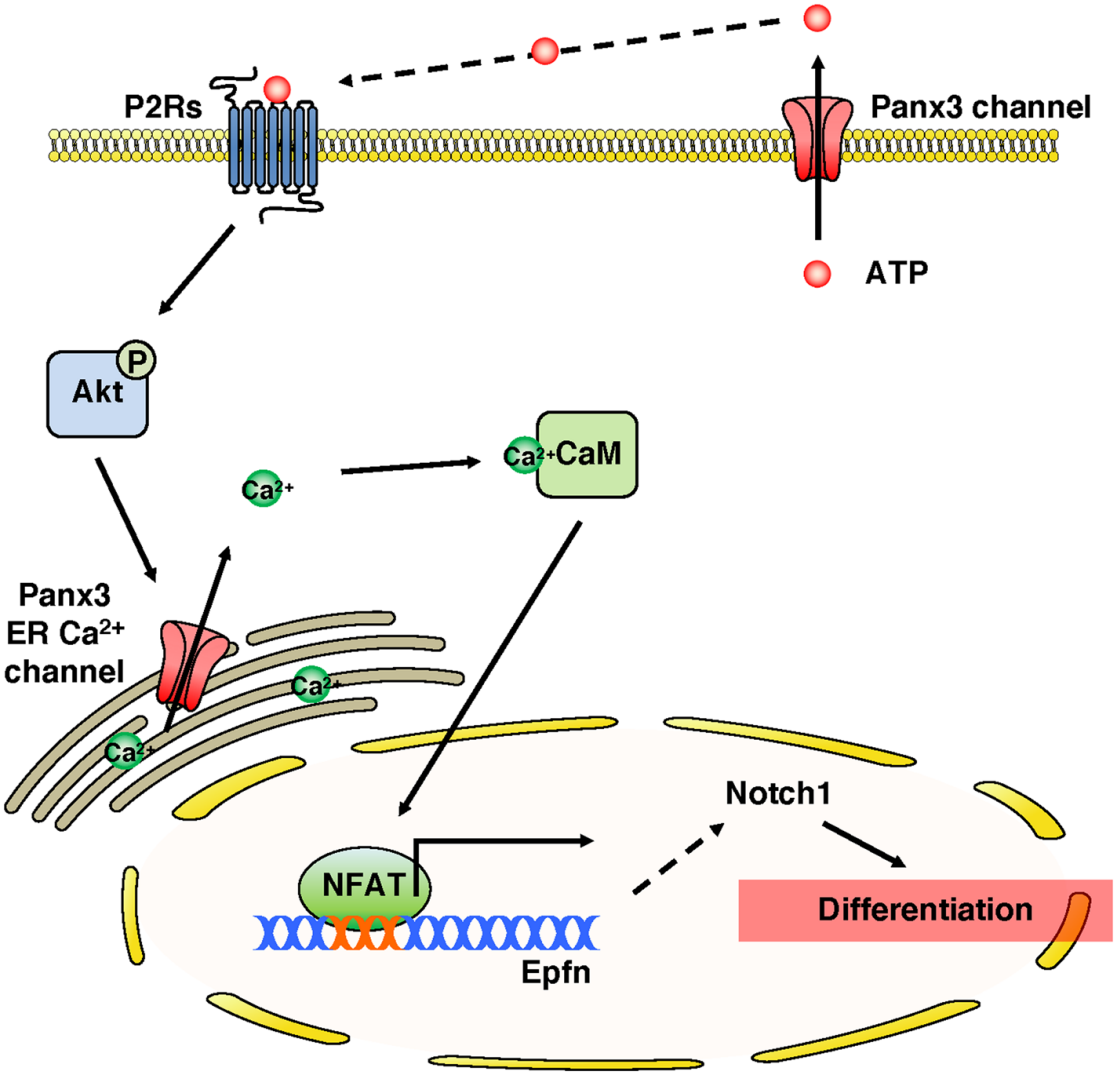
Panx3 signaling pathway in keratinocyte differentiation. Schematic representation of Panx3 signaling pathway in keratinocyte differentiation. 1) ATP releasing Panx3 channel activates Akt signaling in autocrine or paracrine manner. 2) Panx3 ER Ca^2+^ channel opened by Akt signaling promotes calmodulin (CaM)-NFAT signaling by raising up intracellular Ca^2+^ levels. 3) Dephosphorylated NFATc1 (active form NFATc1) enters the nucleus and binds to the promoter regions of Epfn. Epfn then promotes Nocth1 expression.

Here we demonstrated that Panx3 regulates skin development by promoting keratinocyte differentiation via the transcription factor, Epfn. At the tissue level, Panx3^−/−^ mice revealed abnormal skin including less pigmentation, a thinner epidermis and dermis as well as a decrease in hair follicles. At the cellular level, Panx3^−/−^ mice demonstrate decreased Notch1 expression during skin development. In an overexpression cell model, Panx3 promoted keratinocyte cell cycle arrest and led to cell differentiation. Further, Panx3 overexpression promoted Epfn expression during keratinocyte differentiation, while Panx3^−/−^ mice and shPanx3 showed decreased expression. However, Epfn is not crucial to regulate Panx3 expression. Panx3 promotes the activation of NFAT signaling via Panx3 ER Ca^2+^ channel to Epfn expression to enhance Notch1 for keratinocyte differentiation (Fig. 8).

Panx3 is expressed in the epidermis, especially in areas containing actively differentiating keratinocytes in human and murine skin samples^19,26,27^, but the exact mechanisms of how Panx3 regulates skin development has not yet been fully explored. The in vivo and in vitro data presented here demonstrate that Panx3 expression is required to promote keratinocyte differentiation during skin development. Although we did not observe serious skin defects in Panx3^−/−^ mice, the diminished expression of the differentiation marker Notch1 in the skin demonstrates the crucial role of Panx3 in regulating the activities of keratinocytes. The epidermis is composed of five functionally different layers of keratinocytes at different stages of differentiation^3^. From bottom to the upper most are the stratum basal, spinosum, granulosum, lucidum and corneum layers. The deep basal layer contains proliferative epidermal stem cells from which the superficial layers are derived and differentiate. Within these epidermis layers, calcium forms a steep gradient with the highest concentration at the stratum granulosum^28^. This is an indication that keratinocytes require low-level endogenous Ca^2+^ to maintain a basic bioactivity for proliferation and high-level Ca^2+^ for differentiation. Ca^2+^, acting as an important second messenger, is a key regulator of the proliferation, differentiation and morphology in keratinocytes^29,30^. A critical level of cytosolic calcium, achieved either by the influx of extracellular calcium or by intercellular communication via cell–cell contacts, will trigger mechanisms required for initiation of keratinocyte differentiation^31^. Panx3 functions as plasma membrane channel and gap junction which allow the transfer of extracellular small molecules, including Ca^2+^ between cell-extracellular spaces and other cells, respectively. In addition, Panx3 also possesses a unique function as ER Ca^2+^ channel, which can be stimulated to release endogenous Ca^2+^ into cytosol^11,12,14^. Our results confirm that Panx3 has a tight relationship with keratinocyte proliferation and differentiation. In osteoblast differentiation, ATP release through Panx3 activates PI3K/Akt pathway to promote Panx3 ER Ca^2+^ release in an autocrine and paracrine manner. As an ER Ca^2+^ channel, Panx3 is phosphorylated at the Serine 68 residue by Akt signaling, which raises intracellular Ca^2+^ levels and activates other Ca^2+^-dependent signaling pathways including NFAT^18,19^. Here we find that during keratinocyte differentiation, Panx3 functions first as an ATP releasing channel, then as an ER Ca^2+^ channel^19^ and promotes the activation of Akt and NFAT,respectively, similar to osteoblasts. Additonally, we found a change in the cellular morphological characteristics in Panx3 overexpressd HaCaT cells in both KM and KM + high Ca^2+^ (Figs. 3A and 5C). Intracellular Ca^2+^ can regulate cell morphology and the cornification process of epidermal keratinocytes^32,33^. Panx3 mediated intracellular Ca^2+^ signaling pathway also plays a role for cell morphology, although we need further analysis to elucidate the mechanism. Previous study showes that Panx3 overexpression reduced cell proliferation as well as it maintains epidermal architecture in the rat epidermal keratinocytes (REKs) with the epidermal organotypic culture system^26^. Our data was consistent with the previous report about cell proliferation. However, our study can not address more detail Panx3 functions for epidermal architecture with organotypic culture in this article.

During the wound healing process, Panx3^−/−^ mice show delayed epithelialization by inhibiting Wnt and BMP signaling pathways^19^. Wnt/β-catenin signaling promotes keratinocyte proliferation in the hair follicles and in the interfollicular epidermis^34^. Panx3 expression inhibits Wnt/β-catenin signaling in osteoprogenitor proliferation^13^, whereas its overexpression inhibits HaCaT cell proliferation and promotes cell cycle arrest. Although biological evidence of Panx3 for keratinocyte proliferation is similar to that in osteoprogenitor cells, Panx3 function to Wnt/β-catenin signaling in keratinocyte, especially in the case of wound healing is not same. BMP2 promotes Panx3 expression during osteoblast differentiation^14^. As well as Wnt/β-catenin signaling, Panx3 function to BMP signaling in keratinocyte may has differences from that in other cell types. In addition to cell type difference, it is expected that the mechanism in normal keratinocyte differentiation may differ from in the case of wound healing process and requires future experimentation to answer those questions.

Typically, hair follicles regenerate in cyclic bouts consisting of growth (Anagen phase), regression (Catagen phase), and rest (Telogen phase) phases that collectively result in the production of a mature, fully grown hair shaft that extends over the surface of the skin^35^. Hair follicle stem cells in the epithelial niche provide the necessary number and type of specialized cells that are required to construct de novo hair^36^, while the signals are conveyed to the mesenchymal niche to induce dermal papilla growth^37^. The switch between active and quiescent states of hair follicles is a balance between Wnt signal and BMP signal that emanate from the dermal papilla^38^. Calcium channels activity bulge stem cells of the hair follicles cells also contribute to the quiescence-growth cycle^39^. The delayed hair follicle cycling of Panx3^−/−^ mice indicates that Panx3 has a potential role to regulate the activation of epithelial stem cells in hair follicles. We found that hair follicle stem cells were maintained in the quiescent stage and delayed hair follicle regeneration in Panx3^−/−^ mice (data not shown). According to our data, Panx3 may regulate the activation of hair follicle stem cells, but not their maintenance. Adult Panx3^−/−^ mice exhibited impaired de novo production of hair shafts and all temporary hair cell lineages, owing to a prolonged quiescent phase of the first hair cycle.

Epfn was identified by differential hybridization using mRNA isolated from embryonic mouse molars^40^. Epfn and Sp6 are different transcripts generated by alternative promoters from the same gene, but code for the same protein^41^. In contrast to Sp6 expressed ubiquitously in all adult tissues^42^, Epfn is only expressed during mouse embryogenesis in a tissue-specific manner in teeth, skin, hair follicle and limb buds. Besides severe defects in tooth morphology, Epfn^−/−^ mice also display skin abnormalities and hairlessness^20^. It is well documented that Epfn is a key cell cycle regulator of keratinocytes: low levels of Epfn increased the proliferation of HaCaT, while high levels of Epfn promoted cell cycle exit and differentiation^20^. Panx3 overexpression promoted Epfn expression, while Panx3^−/−^ mice and shPanx3 reduced it. However, Epfn^−/−^ mice and siRNA knockdown of Epfn did not demonstrate a change Panx3 expression. These results indicate that Panx3 is an upstream regulator of Epfn involved in keratinocyte differentiation. Unlike the severe skin abnormality of Epfn^−/−^ mice, however, Panx3 ^−/−^ mice do not show obvious skin abnormalities and hairlessness, only displaying a delayed hair cycle and hair regeneration in the early stage of hair growth, suggesting a possible compensation from the other gap junction proteins. A previous study shows that connexin 43 (Cx43) and Panx1 express higher in Panx3^−/−^ mice skin than those in Panx3 ^+/−^ mice skin^19^. However, in our study, Cx43, Panx1 and Panx2 expression were not changed in Panx3^−/−^ mice compared with Panx3^+/−^ mice (Fig. 2A; Supplementary Fig. 6). Thus, other gap junction proteins in skin may help to maintain the metabolism of Panx3^−/−^ skin^43,44^.

Cx43, is ubiquitously expressed in skin and contributes to keratinocyte differentiation^45^. Cx43 mutations directly result in oculodentodigital dysplasia (ODDD) showing the particularly strong abnormalities in the eyes, teeth, and fingers^46^ and rarely demonstrate skin abnormalities. Because Cx43-null mice die at birth due to cardiac malformation, analysis of skin disorders in Cx43-null mice is difficult^47^. According to the studies with Cx43 conditional KO mice and mutant mice, Cx43 function is compensated by other Cxs family members such as Cx26 and Cx30 in keratinocyte differentiation^48–50^. Likewise for osteoblast differentiation, Cx43 expression is regulated by Panx3 via transcription factor, osterix. Cx43 expression is decreased by Panx3^−/−^ mice in bone. On the other hand, Cx43 expression was comparable in skin of Panx3^−/−^ mice. Thus, there are tissue dependent roles between Panx3 and Cx43.

Panx1, a member of the pannexin family, is ubiquitously expressed, especially in the central nervous system^51^. Panx1 is highly expressed in neonatal but not in aged mouse skin and regulates skin development by providing proper skin architecture. Panx1 is required for keratinocyte migration, dermal fibroblast proliferation, and keratinocyte differentiation at early stage, particularly^52^. Panx3 reduces cell proliferation of keratinocytes in the organotypic epidermis^26^. Panx1^−/−^ mice show reduced dermal area, but increased hypodermal thickness in dorsal skin. Furthermore, Panx3 expression is increased in skin of Panx1^−/−^ mice. Thus, the functional differences between Panx1 and Panx3 may compensate for each other, especially at late stages of skin development. In Panx1 and Panx3 double knock-out mouse (dKO), it was shown that neonatal dKO mice have reduced dorsal skin thickness, similar to Panx1^−/^ mice phenotype^43^. According to previous reports and our current results, the compensation mechanism between Panx1 and Panx3 may be due to an age dependency.

Panx3 channels are involved in ATP/Ca^2+^ signaling pathway during osteoblast differentiation in multiple ways^11,14^. First, extracellular ATP released through Panx3 channel activates PI3K-Akt signaling pathway. This in turn leads to the phosphorylation or ER-associated Panx3 to release ER Ca^2+^ store to elevate intracellular Ca^2+^ and activate the calmodulin (CaM)-NFAT pathway. Panx3 mediated NFAT activity promotes osterix (also known as Sp7) transcription to differentiate mature osteoblast^16^. Panx3 also can activate NFAT through its function as an ATP releasing channel as well as its role as an ER Ca^2+^ channel during keratinocyte differentiation^19^. NFAT is essential for maintaining hair follicle stem cell quiescence^53^. and regulates primary keratinocyte cells differentiation in vitro by physical association with Sp1/Sp3^54^. We found Panx3 regulates Epfn/Sp6 expression. Epfn is also upregulated by Ca^2+^. Thus, Ca^2+^ signaling plays a role for Epfn regulation. Actually, Epfn promoter has NFAT binding sequence (data not shown). Thus, Panx3 mediated NFAT signaling can regulate Epfn expression. Besides, Epfn may combine with NFAT directly or indirectly to differentiate cells. Epfn is also reported to promote Notch1 by binding to the Notch1 promoter region^20^. We found that Panx3 ER Ca^2+^ channel strongly regulates Epfn expression. However, it is still uncertain how Panx3 ATP releasing function and gap junction channel regulate keratinocyte differentiation. Blocking of Panx3 ER Ca^2+^ channel function with the Panx3 mutation construct, Ser68Ala, a small amount of Epfn expression still could be observed, but Notch1 expression remained low (Fig. 7A). These results suggest that other Panx3 functions may also be involved. Panx3 acting as an ATP releasing channel reduces intracellular ATP, resulting in a decrease in AMP-activated protein kinase (AMPK) activity and cAMP/PKA signaling in odontoblasts, chondrocytes and osteoblasts^12,13,15^. AMPK regulates Notch1 stability^55^, while PKA is also known to regulate Notch1 during astrocytic differentiation^56^. Thus, it is possible that ATP release through Panx3 can contribute to keratinocyte differentiation. Glycosylated Panx3 can form a membrane channel, but not gap junction^44,57,58^. However, it has also been reported that Panxs can form gap junction channel with the loss of glycosylation^59^. Thus, un-glycosylated Panx3 gap junction channel may contribute keratinocyte differentiaiton. Our results suggest that Panx3 regulates keratinocyte differentiation via NFAT/Ca^2+^ signaling activated by Panx3’s function as an ER Ca^2+^ channel following enhancement of Epfn/Notch1 signaling pathways.

In summary, our data have provided new evidence in keratinocyte differentiation mechanism that Panx3 is an upstream regulator of Epfn via Akt/NFAT signaling pathways by its channel functions.

## Materials and methods

### Animals

Pannexin 3 homozygous (*Panx3*^−/−^), heterozygous mice (*Panx3*^+/−^) mice^16^ and Epiprofin homozygous mice (*Epfn*^−/−^)^40^were generated as described before. Mice were housed under controlled conditions (*i.e.,* temperature, 22 ± 2 °C, relative humidity 65 ± 15%, and 12 h light/dark cycle). All mice had access to a commercial diet and filtered water ad libitum. The animal protocol approved by the NIDCR Animal Care and Use Committee was used for maintaining and handling mice (protocol number ASP15-775). All animals were housed in an animal facility approved by the American Association for the Accreditation of Laboratory Animal Care. For analyzing hair follicle cycling, pair of sibling Panx3^+/−^ and Panx3^−/−^ mice on postborn day 4 (P4), 10 (P10), 20 (P20), 25 (P25) and 38 (P38) were anesthetized with isoflurane. The fur on the dorsal skin was removed with an electric shaver followed by applying VEET gel cream to remove the hair shafts. All experimental procedures in the manuscript were approved by the Animal Care and Use Committee of the National Institute of Dental and Craniofacial Research (protocol no.15-775) and Tohoku University (protocol no. 2017DnA-035), and performed in accordance with relevant guidelines and regulations.

### Cell culture

HaCaT cells were obtained from Silvio Gutkind (UCSD). HaCaT cells were cultured in Keratinocyte-SFM (Gibco, Gaithersburg, MD, USA) with EGF and BPE supplements. For transfection, exponentially growing HaCaT cells were transfected with Panx3 expression vector (pCMV-6 Panx3) (5 μg per 3 × 10^4^ cells) (Origene, Rockville, USA. SC305706) or shRNA plasmid for Panx3 (shPanx3) (Origene, Rockville, USA. TG302692) and their negative controls pCMV6-mock and shControl vector by using a Lipofectamine 3000 Reagent kit (ThermoFisher, Walkersville, USA.) followed by the manufacturer’s protocol^19^. For inhibition of Epfn, HaCaT cells were transfected with siRNA for Epfn (si-Epfn) (Origene, Rockville, USA. SR312895) and their negative control (si-Control) by using a transfection reagent designed for RNAi duplex SiTran1.0 (Origene, Rockville, USA. TT300001) followed by manufacturer’s protocol.

For the cell proliferation assay, HaCaT cells were prepared in 96-well plates at a density of 5 × 10^3^ cells per well for culture. After 12 h, the medium was removed and a Cell Counting Kit (CCK)-8 (Dojindo, Japan) was used according to manufacturer’s instruction to determine the cell numbers based upon reaction product optical density (OD) value. To achieve this, 100 μl of fresh medium containing 10 μl of CCK-8 was added into each well, and the cells were cultured for an additional 1 h, during which time the CCK-8 reagent reacted with cellular metabolic products. The absorbance of the reaction products was then measured with a spectrophotometer at a wavelength of 450 nm using an enzyme-linked immunoadsorbent assay microplate reader (Tecan, USA).

For the cell differentiation assay, HaCaT cells were cultured in normal condition keratinocyte media (KM) with 1.4 mM calcium chloride (Quality Biological. INC. USA) at 37 °C under an atmosphere of 95% air and 5% CO_2_ for three days to induce differentiation.

### Histological analysis

Tissue samples were retrieved at various time points and prepared for histological and immunohistochemical examination. The specimens were fixed overnight in 4% (v/v) Paraformaldehyde Phosphate Buffer Solution (Wako, Japan), embedded in paraffin and then cross-sectioned longitudinally into 5-μm sections using a Leica microtome for Gill’s 3 hematoxylin and aqueous eosin Y solution (H&E) to visualize the overall tissue morphology. All samples were analyzed using an upright microscope, and images were acquired using ScanScope camera (Leica Biosystems)^19^.

### Immunostaining

Tissue sections were deparaffinized in xylene and rehydrated in descending ethanol solution sections. The samples were then immersed in 0.3% H_2_O_2_ to terminate peroxidase activity. After being immersed with antigen retrieval reagent (Dako. S1699) and heated with Decloaking Chamber (Biocare Medical) to improve antigen exposure, sections were applied with 0.1% sodium borohydride (Sigma) at room temperature for 30 min to reduce autofluorescence. Then, sections were blocked with PowerBlock (Biogenex. HK083) for 30 min at room temperature and incubated in a primary antibody solution diluted with antigen diluted reagent with concentration of 1:250, for overnight at 4 °C. Primary antibodies were detected by Alexa Fluor 488 (Invitrogen)-, Cy-3-, or Cy-5 (Jackson ImmunoResearch Laboratories)-conjugated secondary antibody of 1:250 with DAPI (Sigma) 1:1000. Finally, the slides were mounted using a mounting medium (Thermo scientific. TA-030-FM). Five samples from each time point were analyzed manually^19^.

HaCaT cells (1 × 10^4^) were seeded in a 35-mm glass bottom dish and transfected with pCMV6-mock, pCMV6-Panx3, shControl, and shPanx3 vectors, or si-Control, or si-Epfn and cultured in KM for 3 days. The cells were then fixed in 4% Paraformaldehyde Phosphate Buffer Solution (Wako, Japan) + 5% sucrose (MP Biomedicals, France) for 20 min at room temperature followed by 0.1% Triton X-100 (Sigma) for penetration for 5 min and blocked by Power Block (Biogenex) for 30 min. Primary antibodies were applied at 4 °C overnight and were detected by Alexa Fluor 488 (Invitrogen)-, Cy-3-, or Cy-5 (Jackson ImmunoResearch Laboratories)-conjugated secondary antibody of 1:250 with DAPI (Sigma) 1:1000. Immunostaining imaging was performed on a laser scanning A1R MP confocal microscope (Nikon) equipped with plan apo 20 × (NA = 0.75), plan fluor 40 × (NA = 1.30), and plan apo 60 × (NA = 1.4) objectives. Rabbit anti-Panx3 antibody, inhibitory Panx3, and scrambled peptides^15^, and Rabbit anti-Epfn antibody^23^ have been described previously. Mouse anti-connexin 43 (Thermofisher. 138300), Rabbit anti-Notch1 (Cell Signaling Technology. 3608s), Rabbit anti-Ki67 (abcam, ab833Rabbit anti-Filaggrin (Biorbyt. Orb101353) antibodies were used.

ImageJ software (version 2.0.0-rc-69/1.52p, https://imagej.net; provided in the public domain by the National Institutes of Health, Bethesda, MD, USA) is used to analize the quatification of fluorescence intesity. Open figures in ImageJ and turn the RGB figure to RGB stack (Image > Type > RGB stack). Select the stack to work on. Open Threshold (Image > Adjust > Threshold), adjust the threshold to the area of target signals. Use the same setting to evaluate the other figures in the same assay. The fluorescence intensity or the ratio of fluorescence intensity of Panx3^+/−^ to Panx3^−/−^ is evaluated.

For calculating the percentage of Ki67 positive cells, four views of each cell condition were randomly selected. The total number of nuclei and the nuclei with positive Ki67 signals were counted with ImageJ.

### RT-PCR

Total RNA from cells or skin tissues was extracted using the RNeasy Mini Kit and RNase-Free DNase set (Qiagen, USA) according to the manufacturer’s instruction. The RNA was reverse transcribed using the iScript (Bio-Rad.1708840). Total RNA (0.5 μg) was used for reverse transcription to generate cDNA, which was used as a template for PCRs with gene-specific primers. Primers were designed with Mac Vector software (version 17, https://macvector.com/index.html) (Table 1). Real-time PCR amplification was performed with iQ SYBR Green Supermix (Bio-Rad. 64047529) and a C1000 thermocycler (Bio-Rad). Real-time PCR was performed for 45 cycles at 95 °C for 20 s, 60 °C for 20 s, and 72 °C for 20 s. Ribosomal protein S29 was used to normalize gene expression in the samples. All reactions were run in triplicate^19^.

**Table 1:** List of primers for RT-PCR.

Gene name	Forward	Reverse
h S29	CAATATGTGCCGCCAGTGT	GAAGGAAGAGCATTTAGTCCAACT
h Panx3	CGGATAGTCAAGTTCGTAGC	TTACTGGGAGAGAAGCAGC
h K5	GCATCACCGTTCCTGGGTAA	GACACACTTGACTGGCGAGA
h K14	TTGGGGGAGGATATGGTGGT	CAGGCGGTCATTGAGGTTCT
h K1	TTGGTGCTGGTGGTGGATTT	CAAAGCCACCACCACCAAAG
h K10	GACAAAGTTCGGGCTCTGGA	CCCCTGATGTGAGTTGCCAT
h Epfn/Sp6	GCAGCCTCTCCAAACTTACC	TTTCCAGGTCCTCGCAGGTTAC
m S29	GGAGTCACCCACGGAAGTTCGG	GGAAGCACTGGCGGCACATG
m Panx3	TCGCTTAGGGTAGCATTTTCCTC	CATCTTATCCAGGGGCAGTTCC

h: human, m: mouse.

### Flow cytometry analysis

HaCaT cells (1 × 10^6^) were seeded in a 10-cm dish and transfected with pCMV6-mock and pCMV6-Panx3 vectors and cultured in KM for three days. The cells were then collected by centrifugation at 120 × *g* for five min, and fixed with cold 70% ethanol for 24 h followed by washing twice with PBS and staining with PI/RNAse solution (Thermofisher) for 15 min in the dark. The DNA content was analyzed with CellQuest software (http://www.icms.qmul.ac.uk/flowcytometry/flowcytometry/guides/CellQuest%20user%20guide.pdf) on FACSCalibur Station (Becton Dickinson).

### Western blot analysis

HaCaT cells were lysed in Mammalian Cell Lysis Buffer (Abcam. Ab179835) with a halt protease inhibitor cocktail (Thermo Scientific. Rockford IL. USA 78437) on ice. Cells lysis was ultrasonic on ice, done five times with a rotation of 15 s at 1 Hz action and a 15-s stop. Protein concentrations were determined by the BCA kit (Thermofisher. 23225). Ten μg of protein from each group were resolved on sodium dodecyl sulfate (SDS)-polyacrylamide gels (Thermofisher. NP0342) and then transferred onto polyvinyl difluoride membranes (Thermofisher. IB401002). Primary antibodies, Rabbit anti-Epfn^23^, Goat anti-Panx3 (Santa Cruz. sc-51386), Mouse anti-phospho-Akt (Cell Signaling. 4051s), Rabbit anti-Akt antibody (Cell Signaling. 9272), Rabbit anti-phospho-NFAT antibody (Thermofisher. PA5-64484), Mouse anti-NFAT (BD Biosciences. 556602), Mouse anti-Notch1 antibody (Thermofisher. MA5-11961) were used to detect the corresponding proteins. Western blots were developed using SuperSignal West Dura Extended Duration Substrate (Thermo Scientific) and then photographed at Amersham Imager 600 (GE)^19^.

### Statistical analyses

Results are expressed as the mean ± SD. Statistical analysis was performed by the Mann–Whitney U test to compare two groups. For more than two groups, data were analyzed by Kruskal–Wallis test. GraphPad Prism 7 software (https://www.graphpad.com/scientific-software/prism/) was used, in which differences between groups were considered significant when *p* < 0.05 (5%).

## Supplementary Information

Supplementary Figures.
