# Supplementary material for: Pannexin 3 regulates skin development via Epiprofin
Source: Sci Rep. 2021 Jan 19;11:1779. doi: 10.1038/s41598-021-81074-1 (PMC7815752; doi:10.1038/s41598-021-81074-1)
Supplement: Supplementary file 1 — Supplementary Figures. [file 41598_2021_81074_MOESM1_ESM.pdf]

Supplementary Information

Pannexin 3 regulates skin development via Epiprofin

Peipei Zhang[1]*, Masaki Ishikawa[2]*‡, Andrew Doyle[3], Takashi Nakamura[4], Bing He[5], and Yoshihiko Yamada[1⌘]

[1]Molecular Biology Section, National Institute of Dental and Craniofacial Research, National Instistues of Health, Bethesda, MD 20892, USA

[2]Division of Operative Dentistry, Tohoku University Graduate School of Dentistry, Sendai 980-8575, Japan

[3]Cell Biology Section, National Institute of Dental and Craniofacial Research, National Institutes of Health, Bethesda, MD 20892, USA

[4]Division of Molecular Pharmacology and Cell Biophysics, Department of Oral Biology, Tohoku University Graduate School of Dentistry, Sendai 980-8575, Japan

[5]Protein Section, Laboratory of Metabolism, Center for Cancer Research, National Cancer Institute, National Institutes of Health, Bethesda, MD 20892, USA

⌘Deceased

* These authors contributed equally to this work.

‡Address correspondence to: Masaki Ishikawa, Department of Restorative Dentistry, Division of Operative Dentistry, Tohoku University, Graduate School of Dentistry 4-1, Seiryo chou, Aoba-ku, Sendai, Miyagi, 980-8575, Sendai, Japan. Tel: 011-81-22-717-8343; Fax: 011-81-22-717-8344; E-mail: ishikawamas@dent.tohoku.ac.jp.

SUPPLEMENTARY INFORMATION LEGENDS

Supplementary Fig.1. Panx3 regulates hair follicle regeneration during skin development. Representative photographs of Panx3$^{+/-}$ and Panx3$^{-/-}$ mice skin on different postnatal days. Gross appearance of the back skin with shaved hair in each Panx3$^{+/-}$ and Panx3$^{-/-}$ mice.

Supplementary Fig.2. Comparison of Panx3 expression in skin between Panx3$^{+/+}$ and Panx3$^{+/-}$ mice. (A) Semi quantitative RT-PCR band image (a), quantification of semi quantitative RT-PCR band (b), western blot (c), and quantification of protein band (d) of Panx3 expression in skin of 6 weeks old Panx3$^{+/+}$, Panx3$^{+/-}$ and Panx3$^{-/-}$ mice. Ribosomal protein S29 was used as the internal control for RT-PCR. α-Tub: α-Tubulin was used as a loading control for western blot. (B) Representative histology images (H&E staining; upper and middle panel) of Panx3$^{+/+}$ and Panx3$^{+/-}$ mice skin on postnatal day4 (P4). The areas boxed with black dashed lines in upper panels were enlarged below. Ep: epidermis; D: dermis. Measurements of epidermis, dermis thickness and hair follicle numbers in Panx3$^{+/+}$ and Panx3$^{+/-}$ mice on P4 (lower panel). (C) Immunostaining of skin of 6 weeks old Panx3$^{+/+}$ and Panx3$^{+/-}$ mice with antibody to Panx3 (red). The nuclei were counterstained with DAPI (blue in merged). Dotted lines indicate borders between epidermis and dermis. Relative fluorescence intensity comparison of images were analyzed (right panel). *, $P < 0.01$. NS, not significant. Error bars represent the mean ± SD; N = 5.

Supplementary Fig.3. Panx3 expression in Panx3 overexpressed or shPanx3 transfected HaCaT cells. Semi quantitative RT-PCR band images (A) and quantification (B) of Panx3 expression in constructed pCMV6-Panx3 HaCaT (A, 2nd panel; B,a) and shPanx3 HaCaT (A, 4th panel; B,b). pCMV6-mock vector (Mock), pCMV6-Panx3 vector (Panx3), sh scramble RNA (shCon: shControl), and shPanx3 RNA (shPanx3) were transfected into HaCaT cells. Ribosomal protein S29 was used as the internal control. *, $P < 0.01$. Error bars represent the mean ± SD; N = 5.

Supplementary Fig.4. Representative flow cytometry histograms of cell cycle analysis of Panx3 overexpressed HaCaT cells. pCMV6-mock or pCMV6-Panx3 transiently transfected HaCaT cells were cultured in normal condition media for 3 days. The cells were stained with propidium iodide, and cell cycle stages were measured by FACS analysis.

Supplementary Fig.5. Gene expression of proliferation and differentiation markers in KM + high Ca^{2+} condition. (A) qPCR for Panx3, Filaggrin, K10, and K14 in HaCaT cells cultured in KM or KM + high Ca^{2+}. (B) qPCR for Panx3 and Notch1 in shControl (shCon) and shPanx3 transfected HaCaT cells. *, $P < 0.01$. Error bars represent the mean ± SD; N = 3.

Supplementary Fig.6. Gene expression of other Pannexin family in skin. Quantitative RT-PCR of Panx1 and Panx2 expression using total RNA prepared from skin of 6 weeks old

$Panx3^{+/-}$ and $Panx3^{-/-}$ mice. Results represent the mean ± SD; N = 3. *, NS, not significant.

Ribosomal protein S29 was used as the internal control.

Full Original Blots-I.

These figures display the full original blots for Fig.6C,a shown in the text/Results. The identification of bands was based on the expected molecular weight. For details see legend of Fig.6. Rectangle delimited areas were presented in Fig.6C,a of the manuscript.

Full Original Blots-II.

These figures display the full original blots for Fig.6D,a shown in the text/Results. The identification of bands was based on the expected molecular weight. For details see legend of Fig.6. Rectangle delimited areas were presented in Fig.6D,a of the manuscript.

Full Original Blots-III.

These figures display the full original blots for Fig.7A,a shown in the text/Results. The identification of bands was based on the expected molecular weight. For details see legend of Fig.7. Rectangle delimited areas were presented in Fig.7A,a of the manuscript.

Full Original Blots-IV.

These figures display the full original blots for Fig.7B,a shown in the text/Results. The identification of bands was based on the expected molecular weight. For details see legend of Fig.7. Rectangle delimited areas were presented in Fig.7B,a of the manuscript.

Full Original Blots-V.

These figures display the full original gel images and blots for Supplementary Fig.2A,a and c shown in the text/Results. The identification of bands was based on the expected molecular

weight. For details see legend of Supplementary Fig.2. Rectangle delimited areas were presented in Supplementary Fig.2A,a and c of the manuscript.

Full Original Blots-VI.

These figures display the full original blots for Supplementary Fig.3A shown in the text/Results. The identification of bands was based on the expected molecular weight. For details see legend of Supplementary Fig.3A. Rectangle delimited areas were presented in Supplementary Fig.3A of the manuscript.

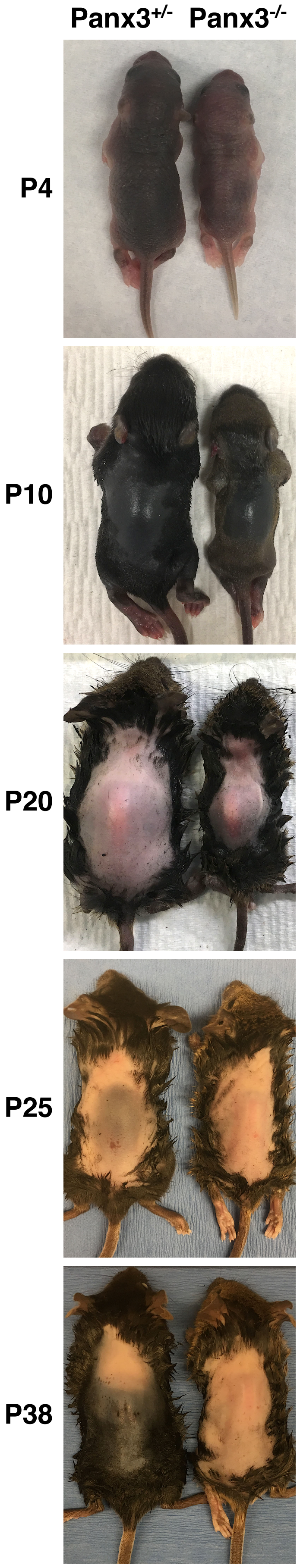

Panx3+/- Panx3-/-
P4
P10
P20
P25
P38

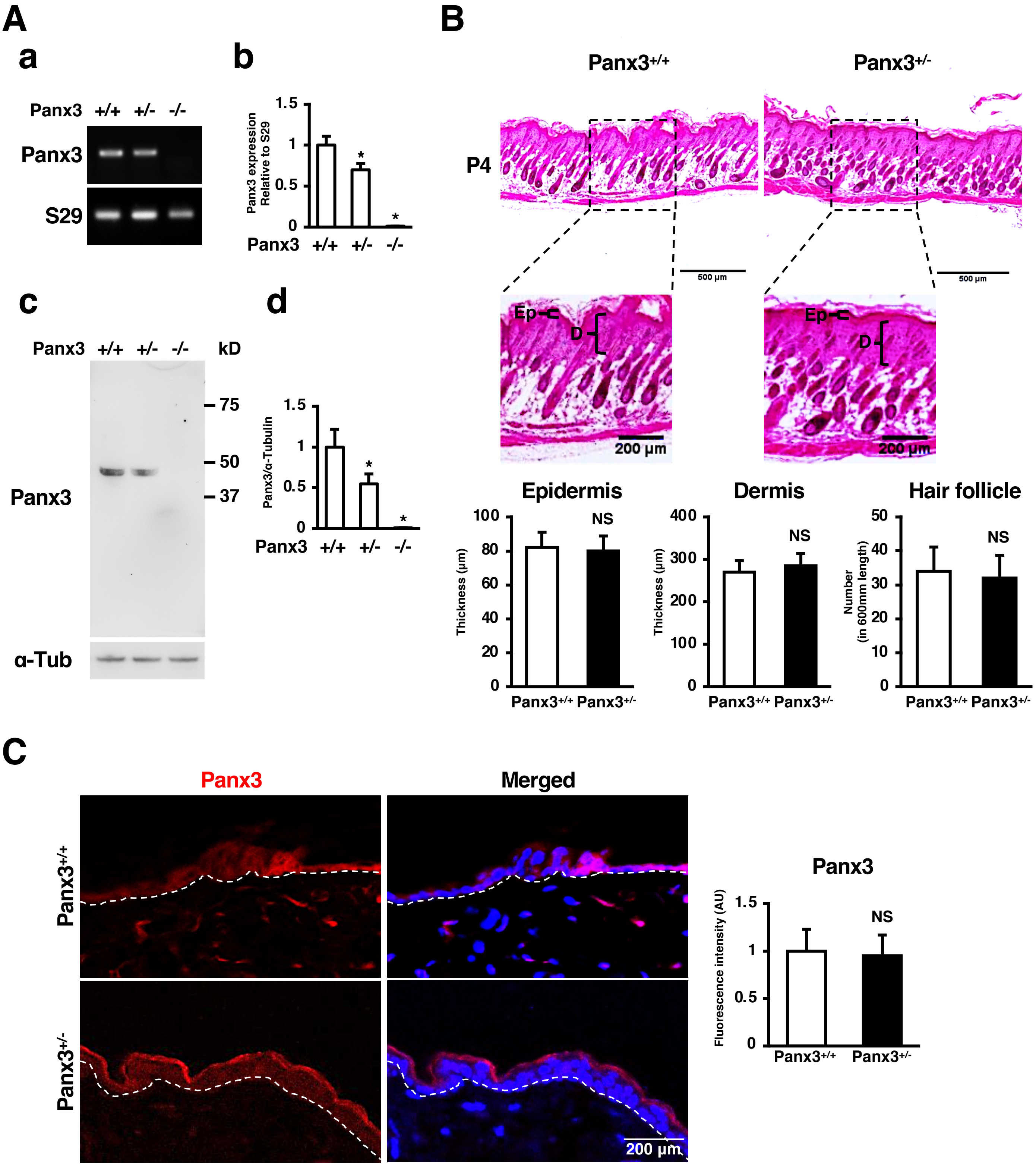

A
a
Panx3 +/+ +/- -/-
Panx3
S29
b
Panx3 expression Relative to S29
1.5
1
0.5
0
Panx3 +/+ +/- -/-
c
Panx3 +/+ +/- -/- kD
75
50
37
Panx3
α-Tub
d
Panx3/α-Tubulin
1.5
1
0.5
0
Panx3 +/+ +/- -/-
B
Panx3+/+
Panx3+/-
P4
500 μm
Ep
D
200 μm
Epidermis
Dermis
Hair follicle
Thickness (μm)
Number (in 600mm length)
NS
Panx3+/+ Panx3+/-
C
Panx3
Merged
Panx3+/+
Panx3+/-
200 μm
Fluorescence intensity (AU)

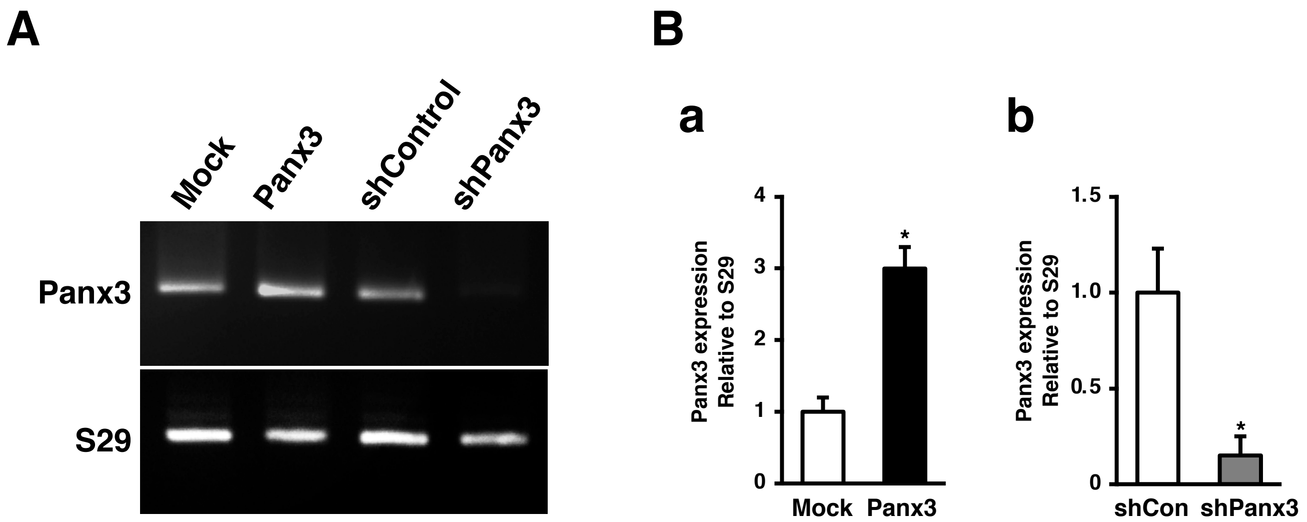

A
Mock
Panx3
shControl
shPanx3
Panx3
S29
B
a
b
Panx3 expression
Relative to S29
0
1
2
3
4
*
Mock
Panx3
Panx3 expression
Relative to S29
0
0.5
1.0
1.5
*
shCon
shPanx3

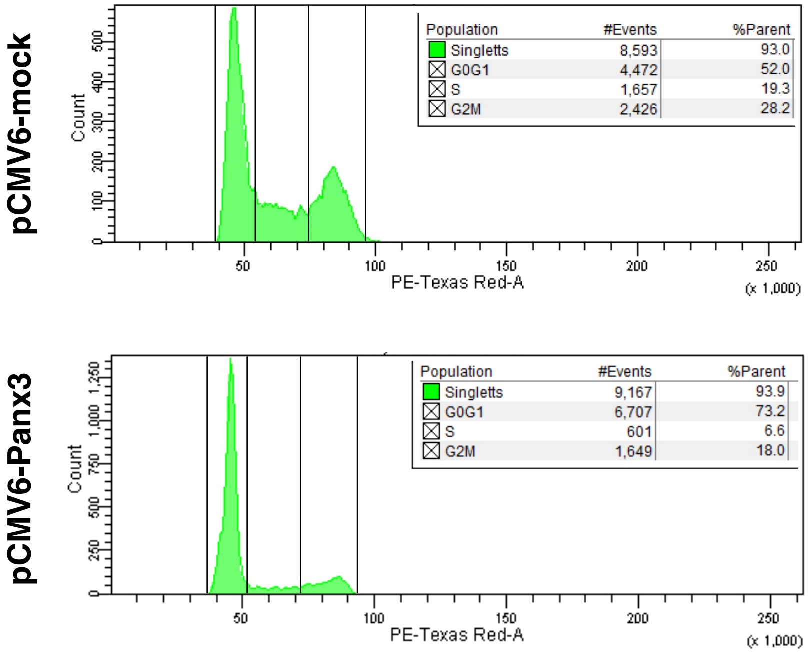
pCMV6-mock
Count
PE-Texas Red-A
(x 1,000)
Population #Events %Parent
Singletts 8,593 93.0
G0G1 4,472 52.0
S 1,657 19.3
G2M 2,426 28.2
pCMV6-Panx3
Count
PE-Texas Red-A
(x 1,000)
Population #Events %Parent
Singletts 9,167 93.9
G0G1 6,707 73.2
S 601 6.6
G2M 1,649 18.0

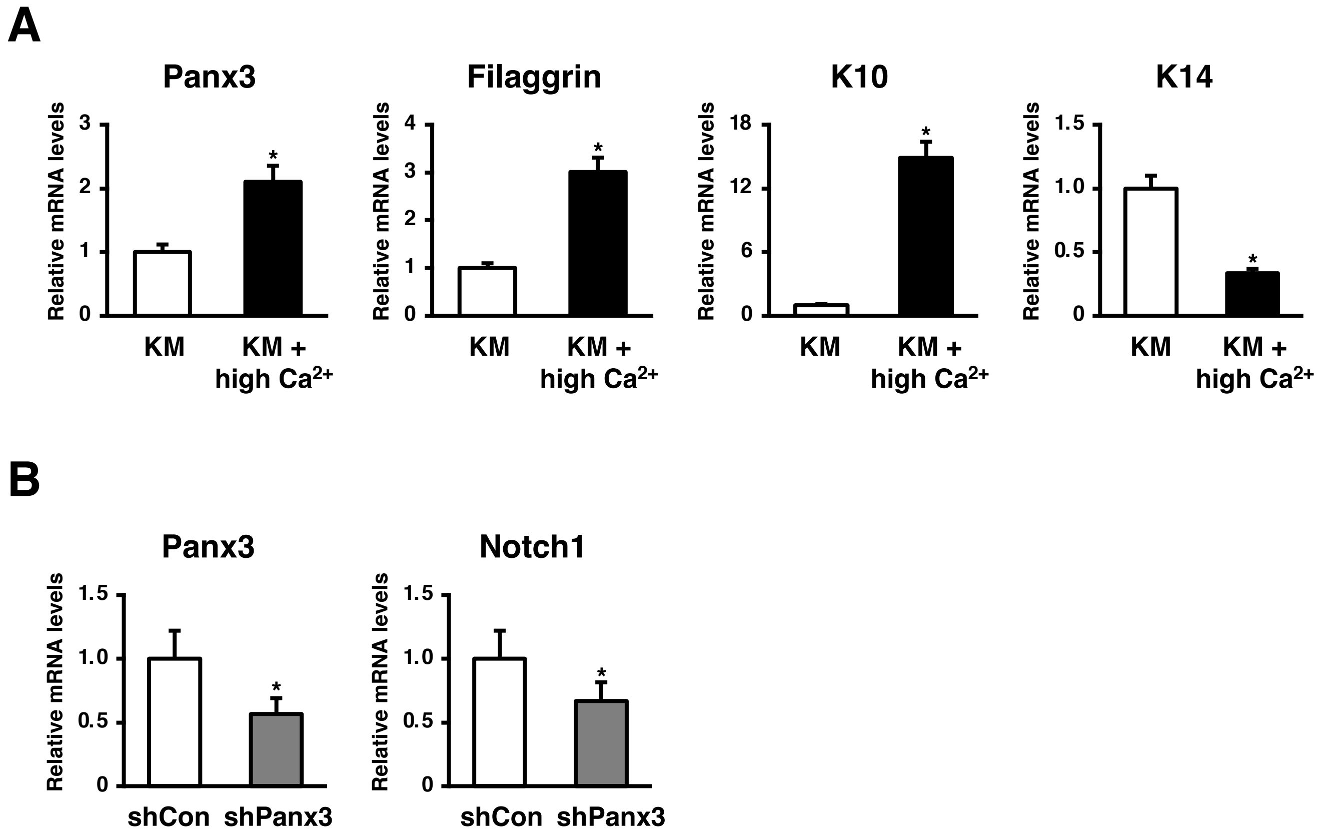
A
Panx3
Filaggrin
K10
K14
Relative mRNA levels
KM
KM + high Ca2+
B
Panx3
Notch1
shCon
shPanx3

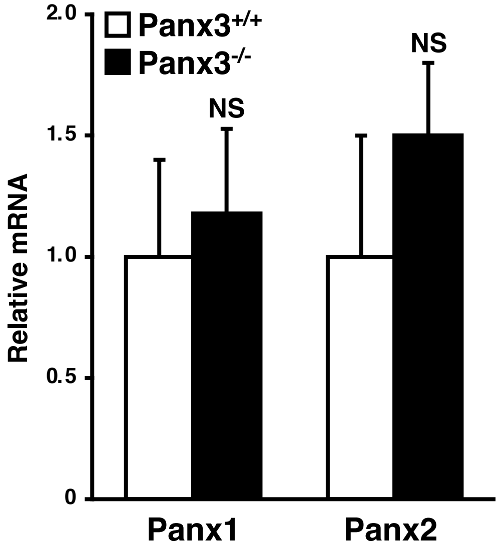

Panx3+/+
Panx3-/-
NS
NS
2.0
1.5
1.0
0.5
0
Relative mRNA
Panx1
Panx2

Panx3

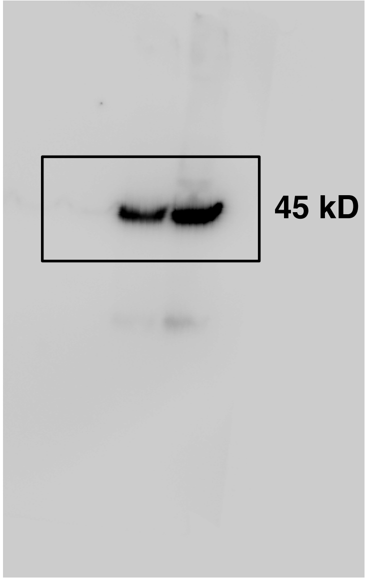

45 kD

Epfn

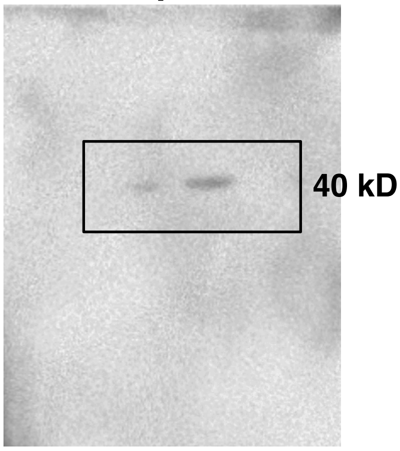

40 kD

Notch1

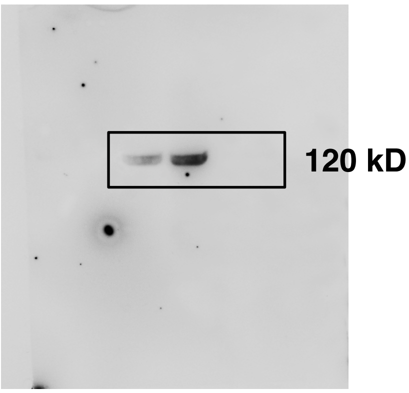

120 kD

α-Tubulin

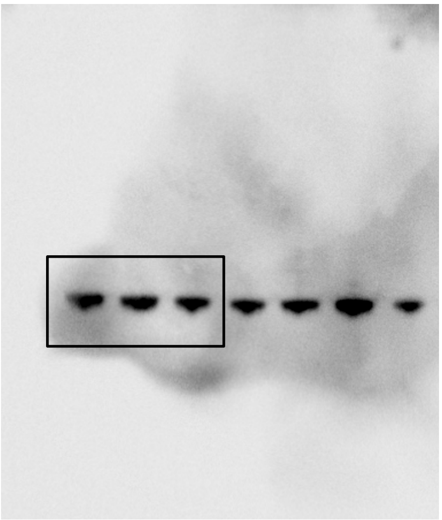

50 kD

Panx3

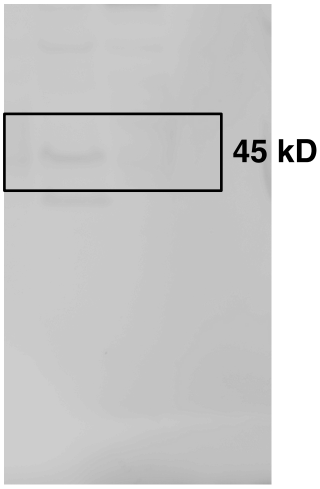

Epfn

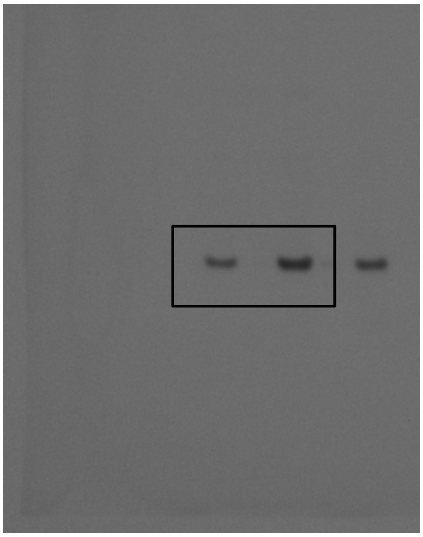

Notch1

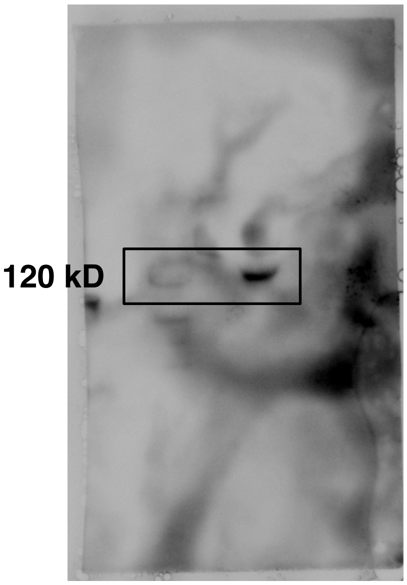

α-Tubulin

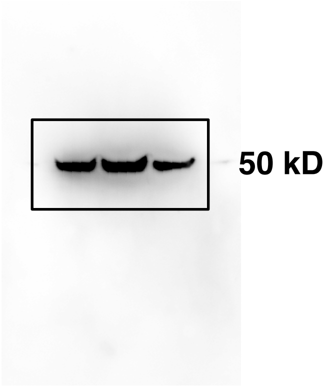

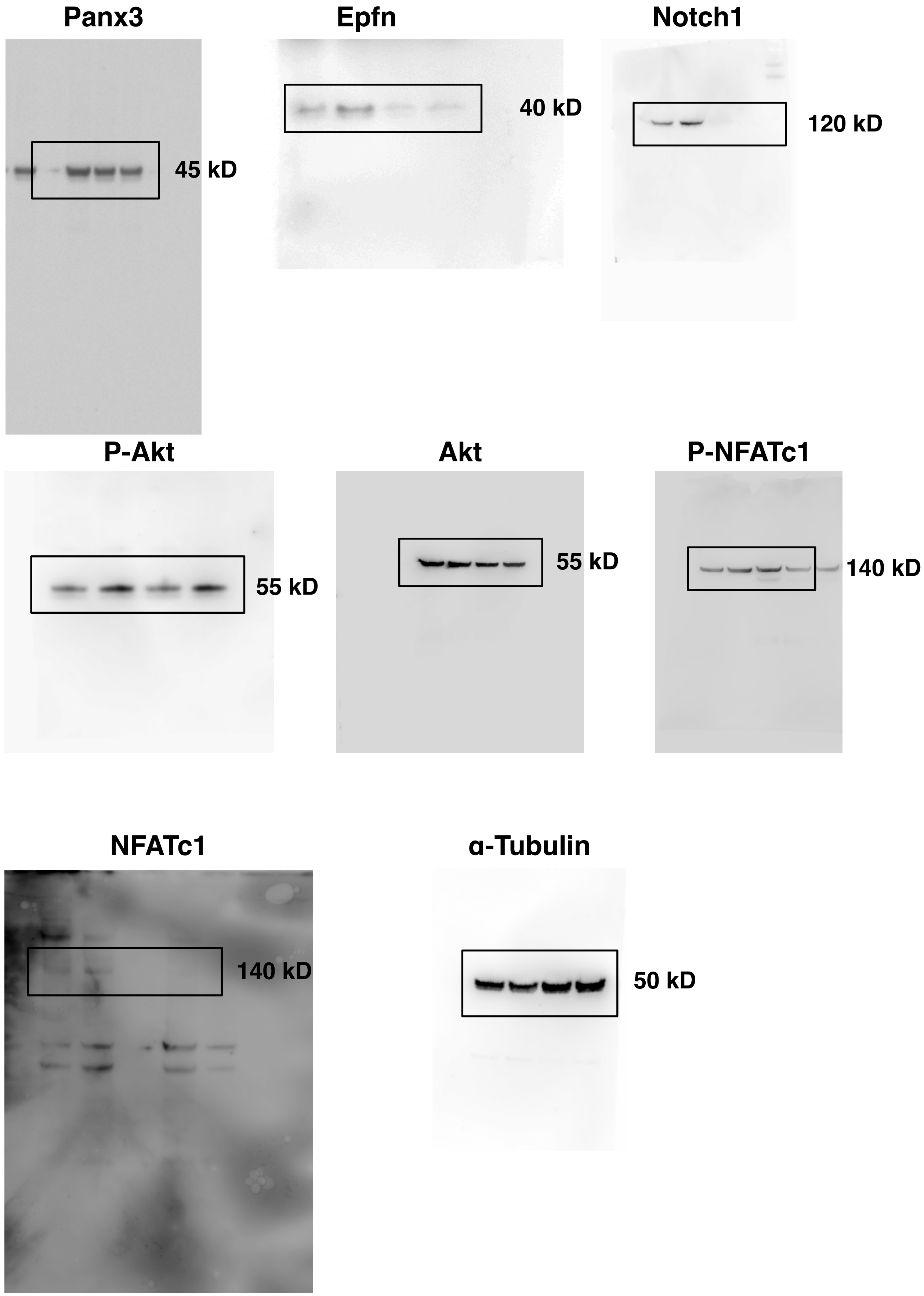
Panx3
45 kD
Epfn
40 kD
Notch1
120 kD
P-Akt
55 kD
Akt
55 kD
P-NFATc1
140 kD
NFATc1
140 kD
α-Tubulin
50 kD

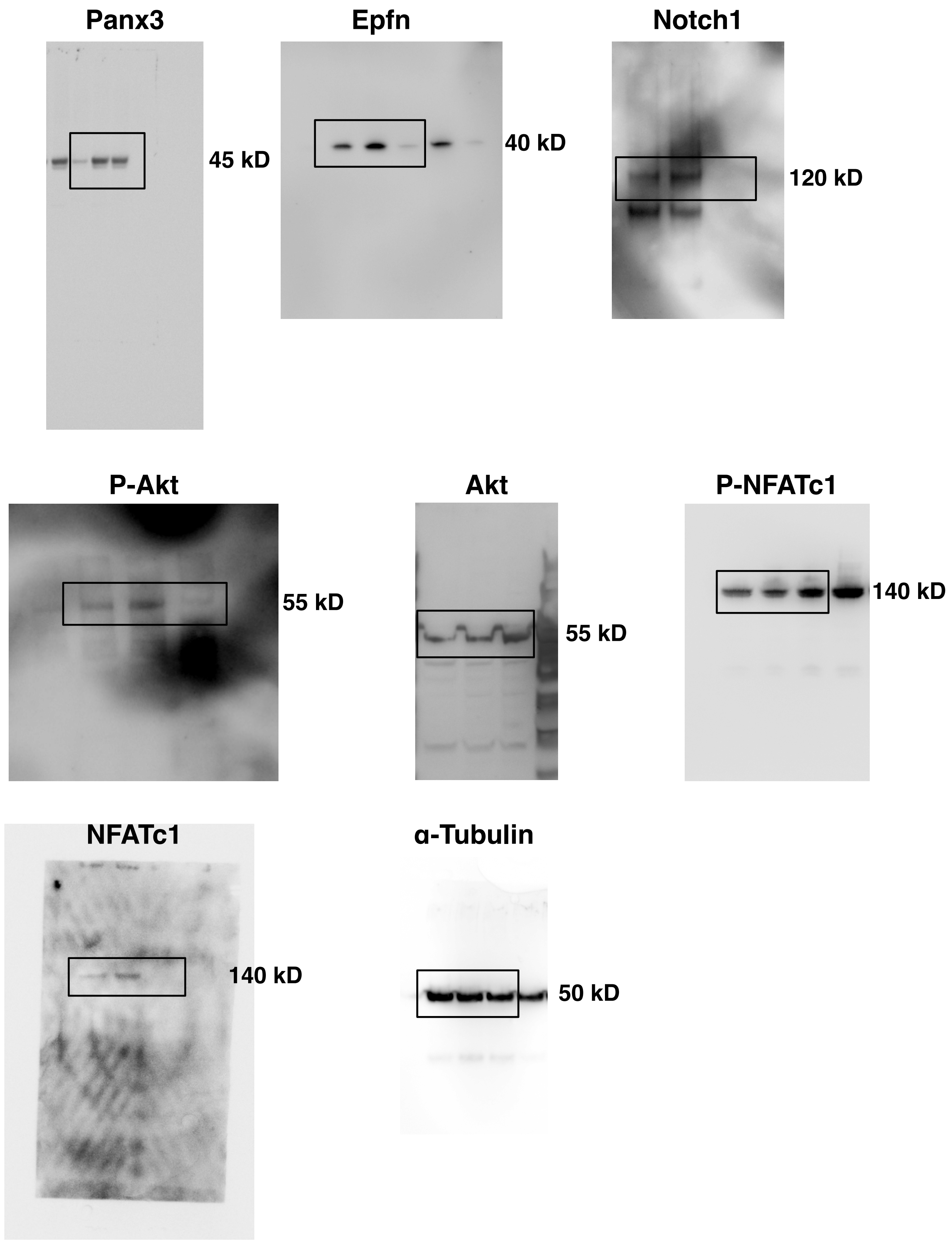
Panx3
45 kD
Epfn
40 kD
Notch1
120 kD
P-Akt
55 kD
Akt
55 kD
P-NFATc1
140 kD
NFATc1
140 kD
α-Tubulin
50 kD

Panx3

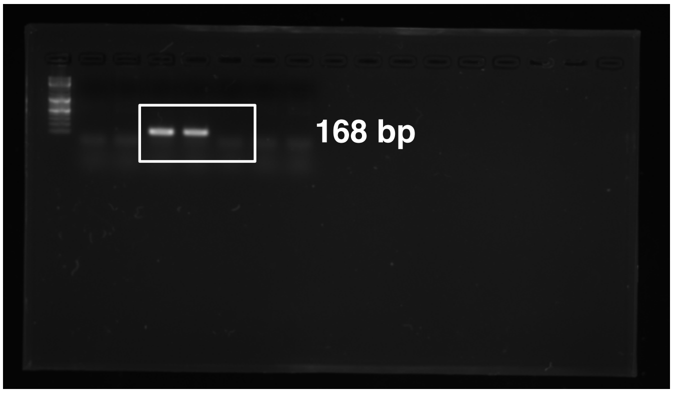

S29

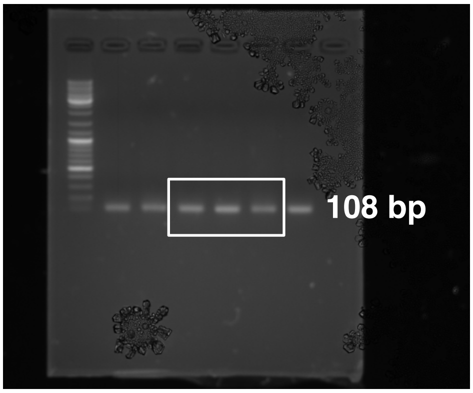

Panx3

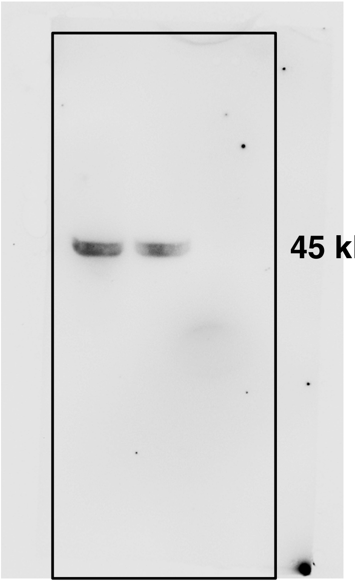

45 kD

α-Tubulin

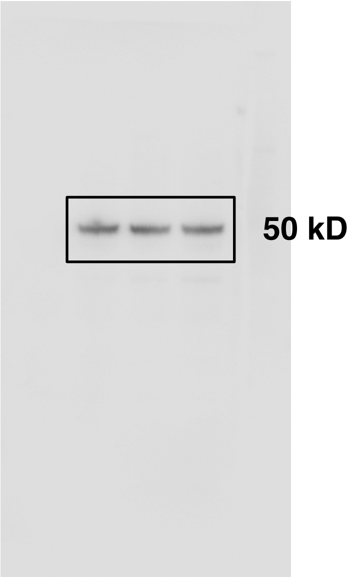

50 kD

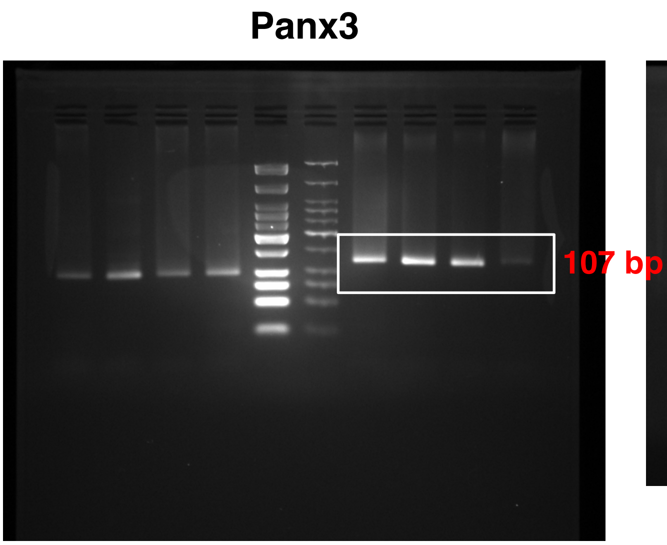
Panx3
107 bp

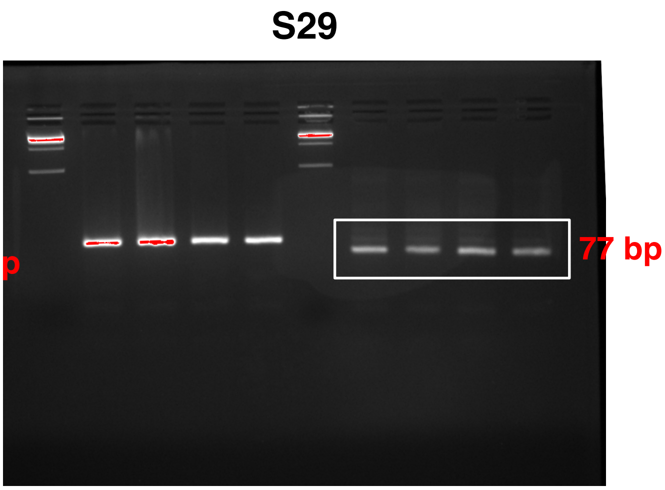
S29
77 bp
